# Supplementary material for: Room temperature catalytic upgrading of unpurified lignin depolymerization oil into bisphenols and butene-2
Source: Nat Commun. 2024 Jul 13;15:5892. doi: 10.1038/s41467-024-49812-x (PMC11246530; doi:10.1038/s41467-024-49812-x)
Supplement: Supplementary file 1 — Supplementary Information [file 41467_2024_49812_MOESM1_ESM.pdf]

Supplementary Information for

Room temperature catalytic upgrading of unpurified lignin depolymerization oil into bisphenols and butene-2

Elena Subbotina, Layra Rodrigues Souza, Julie Zimmerman, Paul Anastas
Corresponding authors email: paul.anastas@yale.edu; elenasu@kth.se

Table of Content

This PDF file includes:

Supplementary Figs. 1 to 39
Supplementary Tables 1 to 2
Supplementary References 1 to 2

Supplementary Information

1. Experimental procedures

Preparation of propenyl syringol (PS)

A dry flask equipped with a dry stir bar was filled with 33 mg (0.0346 mmol, 0.57 mol%) of carbonyl-chloro-hydrido-tris-(triphenylphosphine)-ruthenium(II) (RuH) followed by 1.18 g (6 mmol) of allylsyringol in glovebox. The reaction flask was closed with a septum and placed in a sand bath preheated to 60 °C. The reaction was monitored by ^{1}H NMR. Upon the completion of the reaction (12 hours) the product was isolated from the crude reaction mixture via column chromatography (hexane/EtOAc = 85:15). PS was obtained in 75% yield. ^{1}H NMR spectrum corresponds to previously reported (Supplementary Fig. 38).[1]

Preparation of methyl cinnamyl ether

In a glovebox 307 mg (7.67 mmol) of NaH (60 wt% in mineral oil) was added into a dry flask, equipped with a dry stir bar, followed by the addition of anhydrous THF (10 mL). The mineral oil was removed by an extraction with THF as follows. The mixture was stirred for 10 minutes, after which the stirring was turned off and the precipitate was allowed to settle. The supernatant was removed with a pipette. The procedure was repeated 2 more times. A fresh portion of anhydrous THF (10 mL) was added into the flask followed by a dropwise addition of the solution of cinnamyl alcohol (415.1 mg, 3.1 mmol) in 5 mL THF. The reaction mixture was allowed to stir for 30 minutes. After that 0.579 mL (11.58 mmol) of methyl iodide was added dropwise. The reaction was left under stirring for 16 hours. Upon completion the reaction mixture was taken out of the glovebox and carefully quenched with water. When evolution of hydrogen gas ceased the product was extracted with EtOAc. The organic layers were combined, dried under anhydrous Na_2SO_4, the solvent was removed under reduced pressure, and the final product was dried under vacuum (67% yield). ^{1}H NMR spectrum corresponds to previously reported (Supplementary Fig. 39).[2]

^{1}H NMR (400 MHz, $CDCl_3$) δ 7.41 – 7.18 (m, 5H), 6.60 (dt, J = 15.9, 1.6 Hz, 1H), 6.27 (dt, J = 15.9, 6.0 Hz, 1H), 4.08 (dd, J = 6.0, 1.6 Hz, 2H), 3.38 (s, 3H).

Metathesis of IE catalyzed by HGII in the presence of RuH

The reaction was performed following the general procedure described in Methods section of the manuscript, but with an addition of a corresponding amount of RuH as a solution in DCM. When the self-metathesis of IE was performed using RuH/HGII mixture (0.01 mol% HGII as a solution in toluene + 0.015 mol% RuH as a solution in DCM) only 21% conversion of IE was achieved after 3h, and 30% after more than a day. However, when the RuH/HGII molar ratio was lowered to 0.9, the reaction was completed within 1 hour. The exact reason for this observation is unclear, however, in the presence of RuH IE may get involved in a non-productive hydrometalation/reductive elimination pathway, which in turn can slow down the metathesis.

2. Supplementary Figures

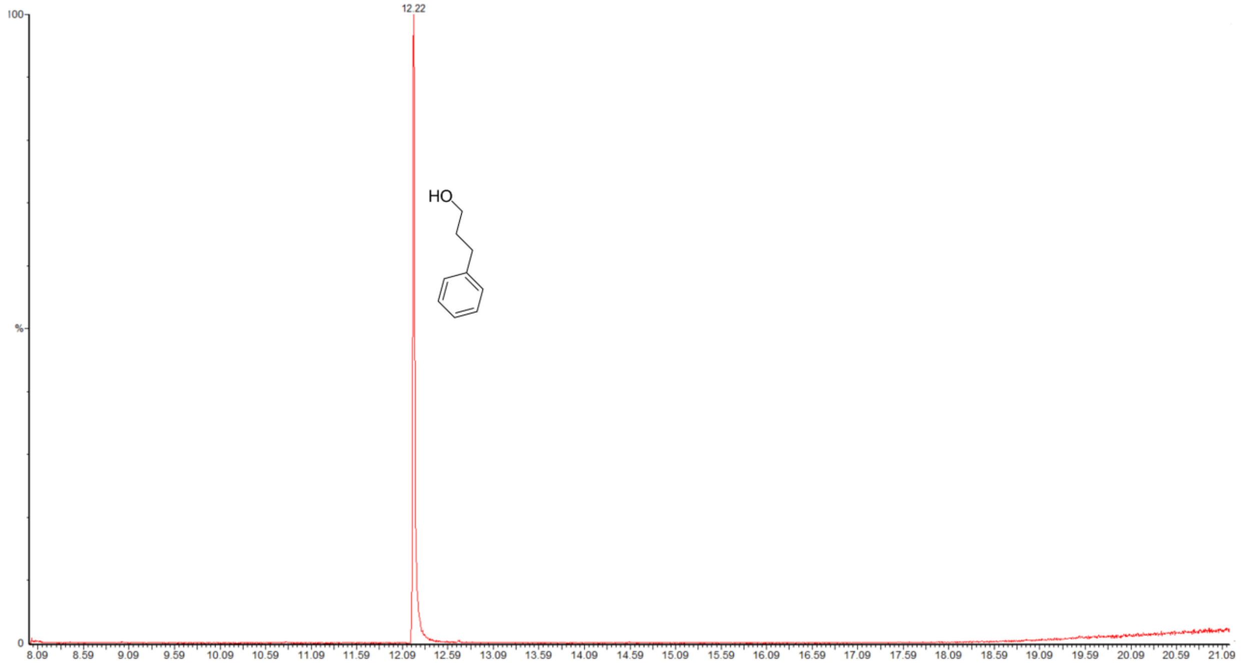

Retention time, mins

Supplementary Fig.1. GC-MS chromatogram of the reaction mixture of metathesis of IE in the presence of 20 mol% of 3-Phenyl propanol.

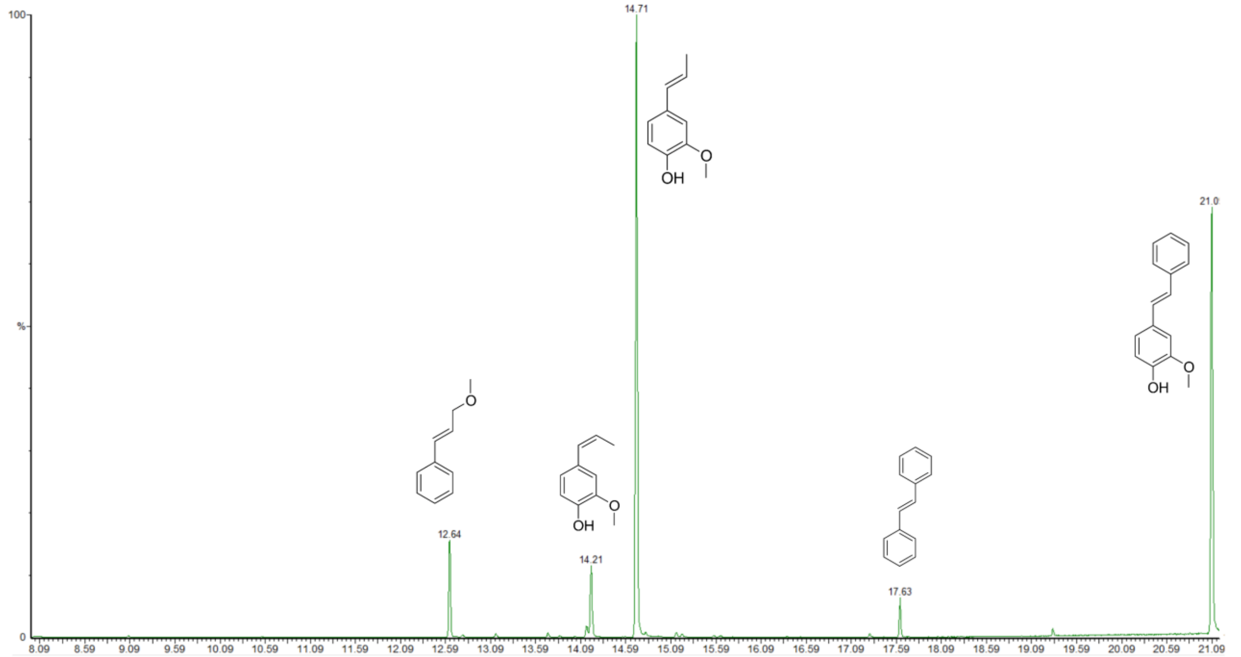

Retention time, mins

Supplementary Fig.2. GC-MS chromatogram of the reaction mixture of the metathesis of IE in the presence of 20 mol% of Methyl cinnamyl ether.

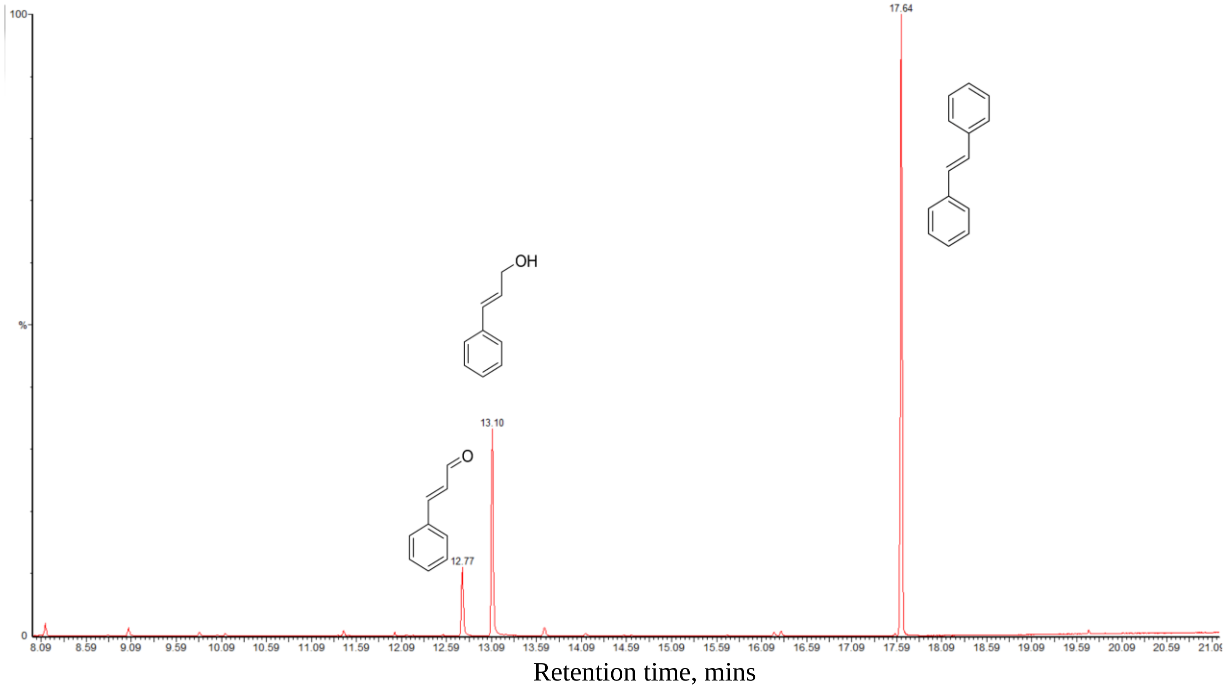

Supplementary Fig.3. GC-MS chromatogram of the reaction mixture containing cinnamyl alcohol and 4 mol% HGII in toluene-d8 (3 days).

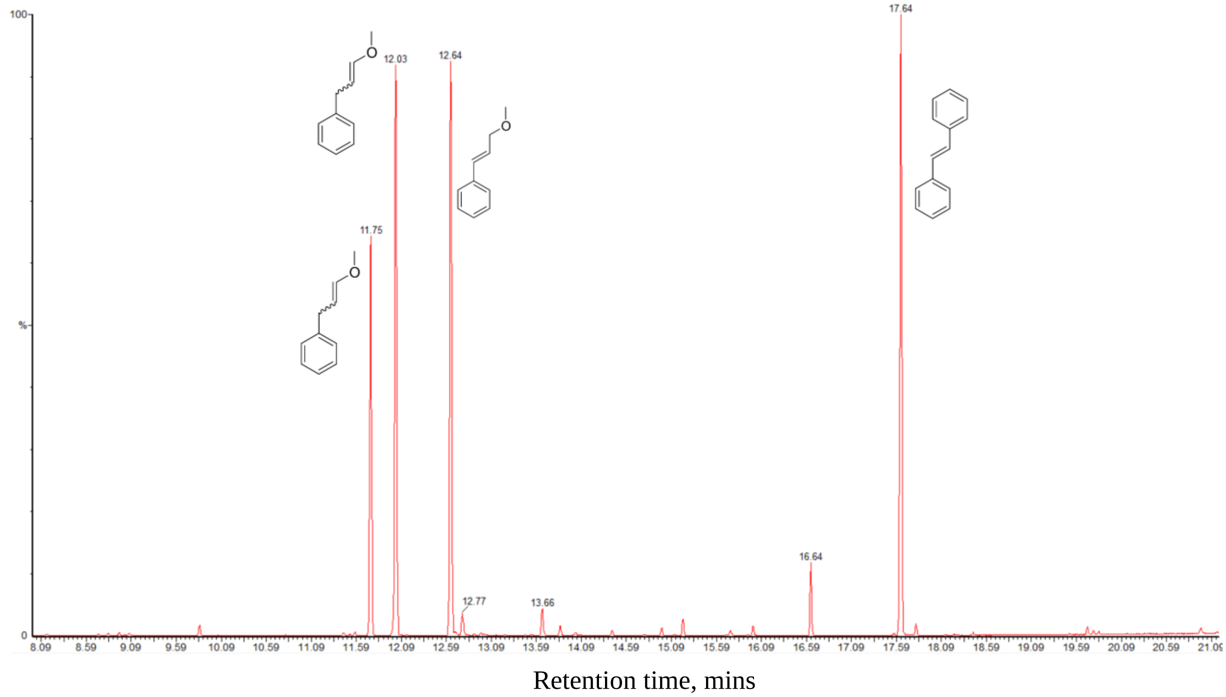

Supplementary Fig.4. GC-MS chromatogram of the reaction mixture containing methyl cinnamyl ether and 4 mol% HGII toluene-d8 (3 days).

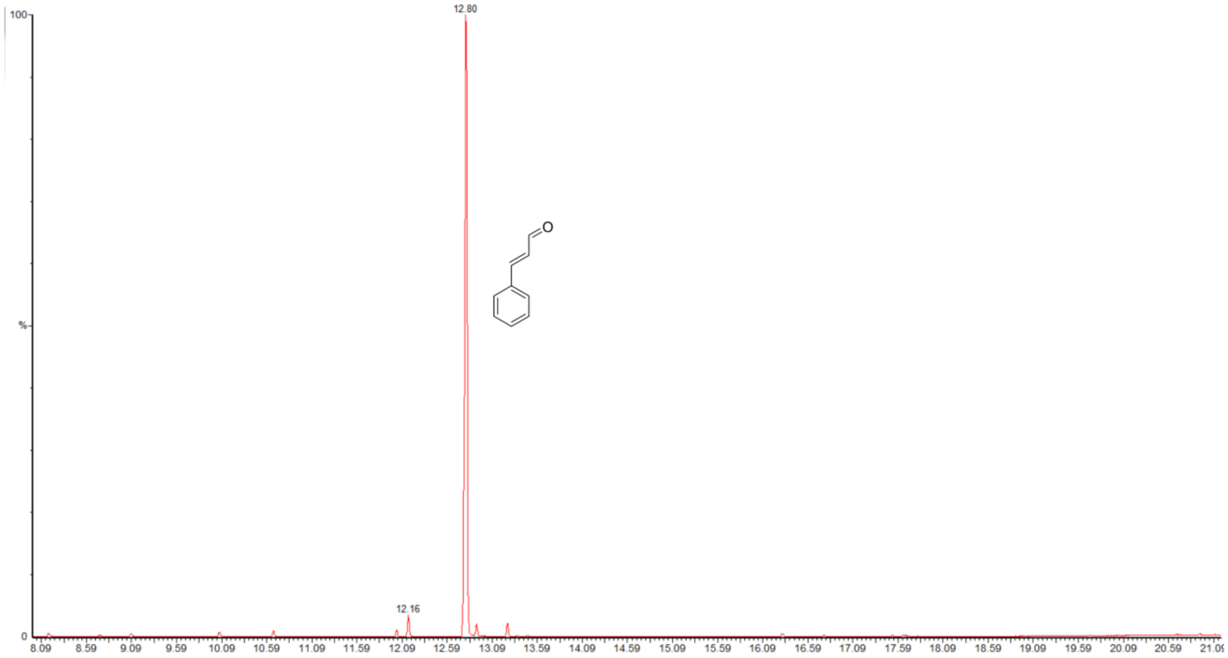

Retention time, mins

Supplementary Fig.5. GC-MS chromatogram of the reaction mixture containing cinnamyl aldehyde and 4 mol% HGII in toluene-d8 (3 days).

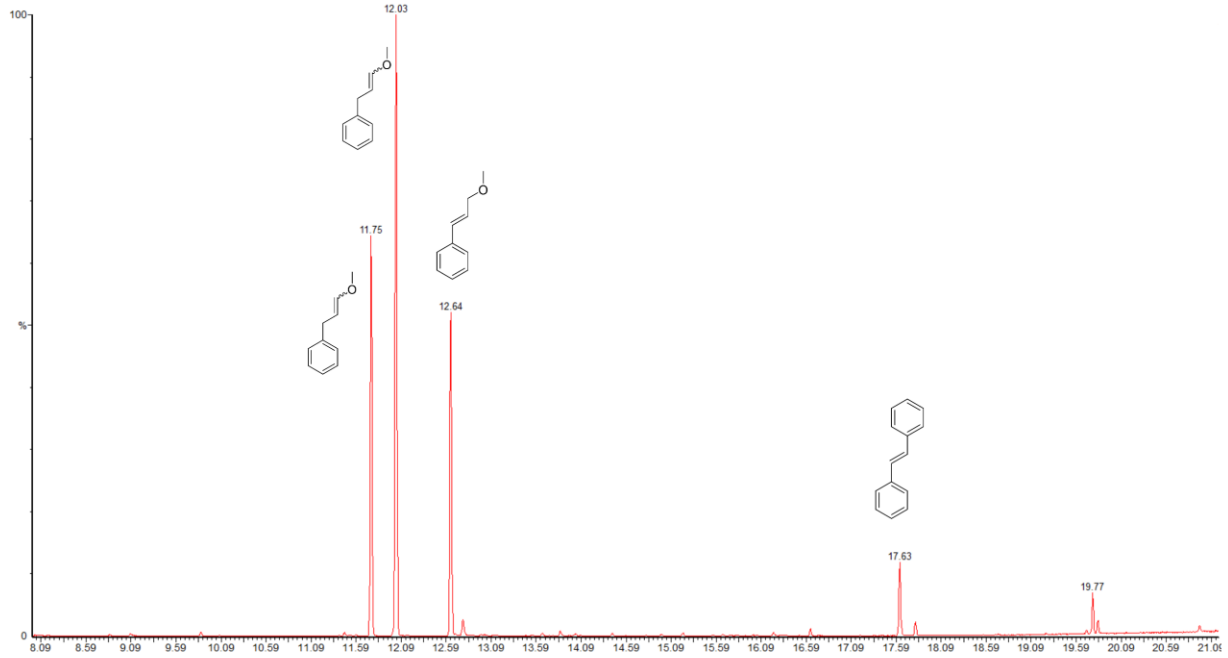

Retention time, mins

Supplementary Fig.6. GC-MS chromatogram of the reaction mixture containing methyl cinnamyl ether and 4 mol% HGII in toluene-d8 after 1 hour at 110 °C.

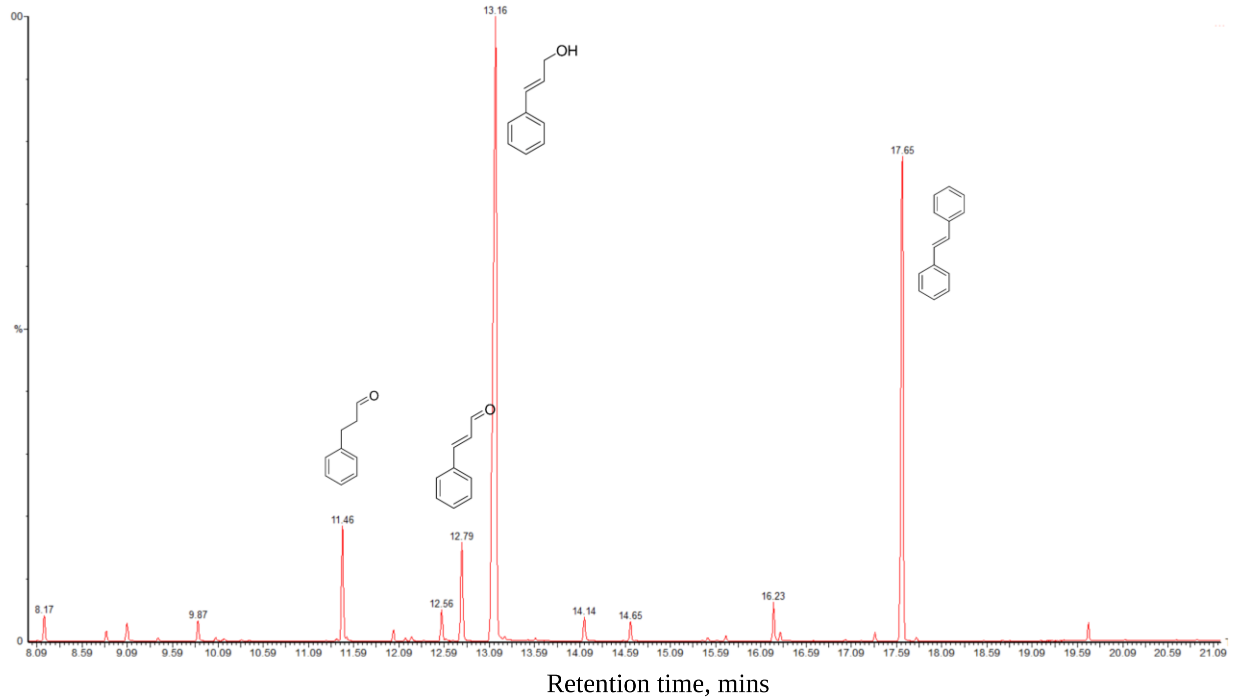

Supplementary Fig.7. GC-MS chromatogram of the reaction mixture containing cinnamyl alcohol and 4 mol% HGII in toluene-d8 after 1 hour at 110 °C.

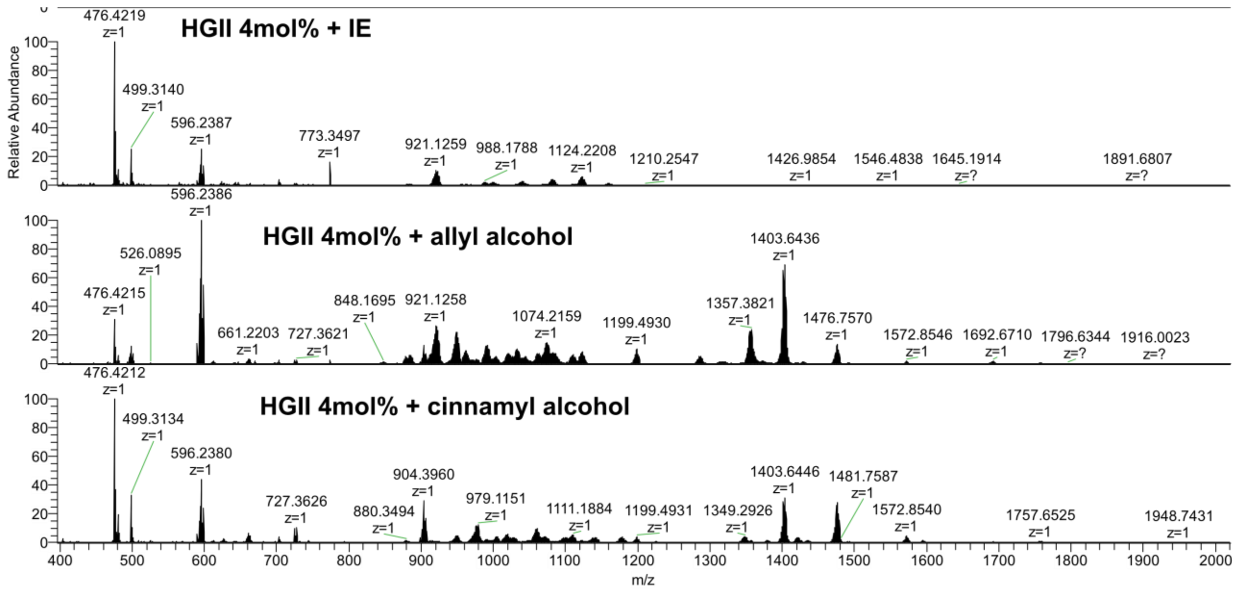

Supplementary Fig.8. ESI-HRMS spectra for the reaction mixtures containing (from top to bottom): HGII 4 mol% + IE, HGII 4 mol% + allyl alcohol, HGII 4 mol% + cinnamyl alcohol.

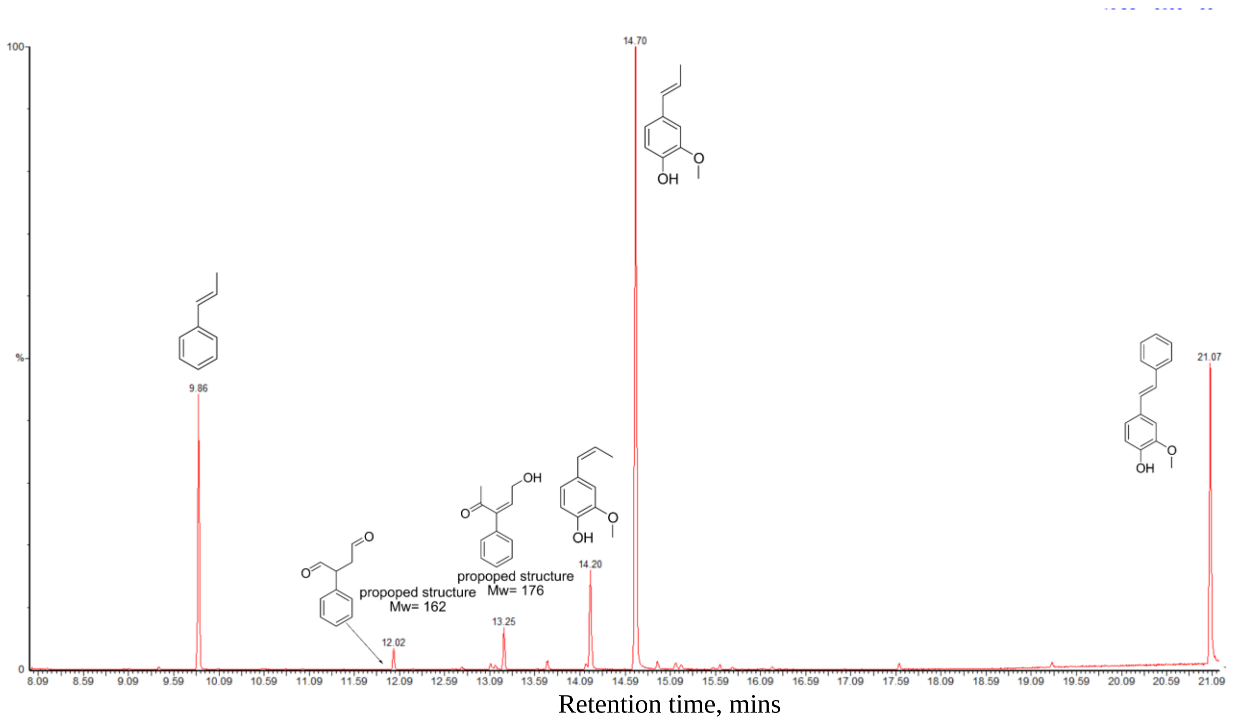

Supplementary Fig.9. GC-MS chromatogram of the reaction mixture of metathesis of IE in the presence of 5 mol% of cinnamyl alcohol with a single portion addition of HGII (0.5 mol%).

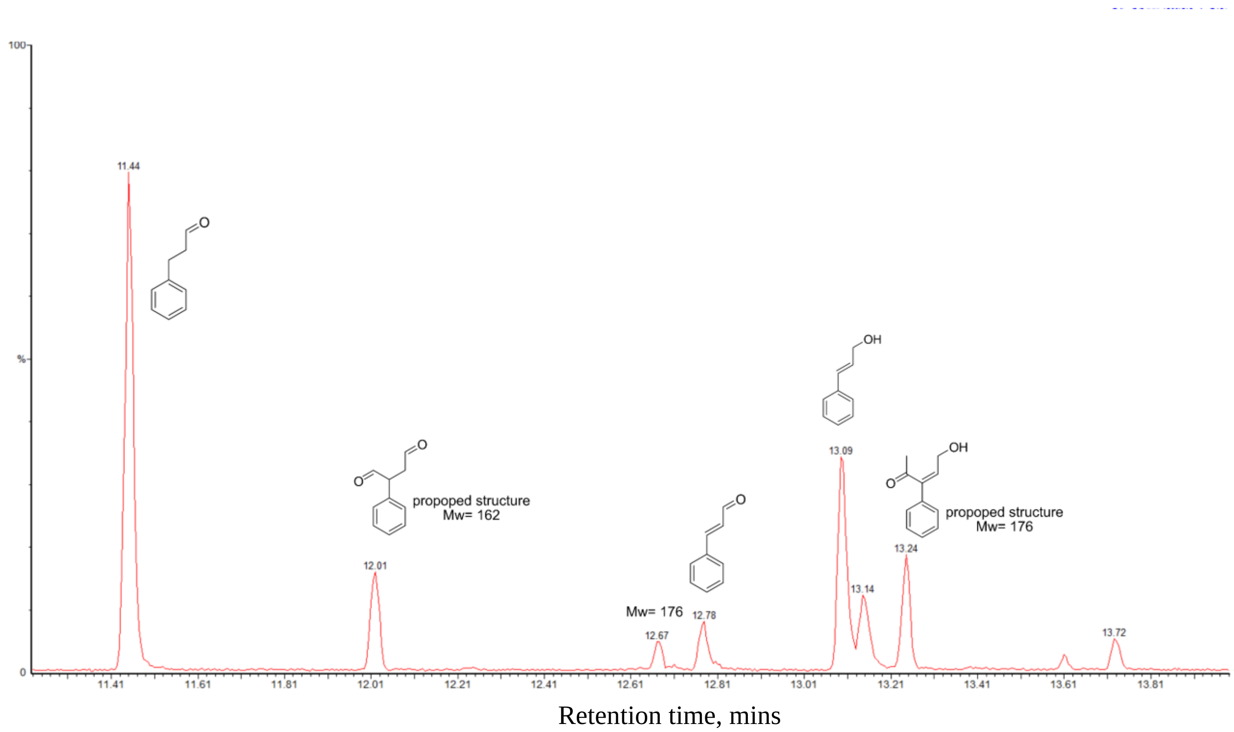

Supplementary Fig.10. GC-MS chromatogram of the reaction mixture of metathesis of IE in the presence of 5 mol% of cinnamyl alcohol with a stepwise addition of HGII up to 0.5 mol%.

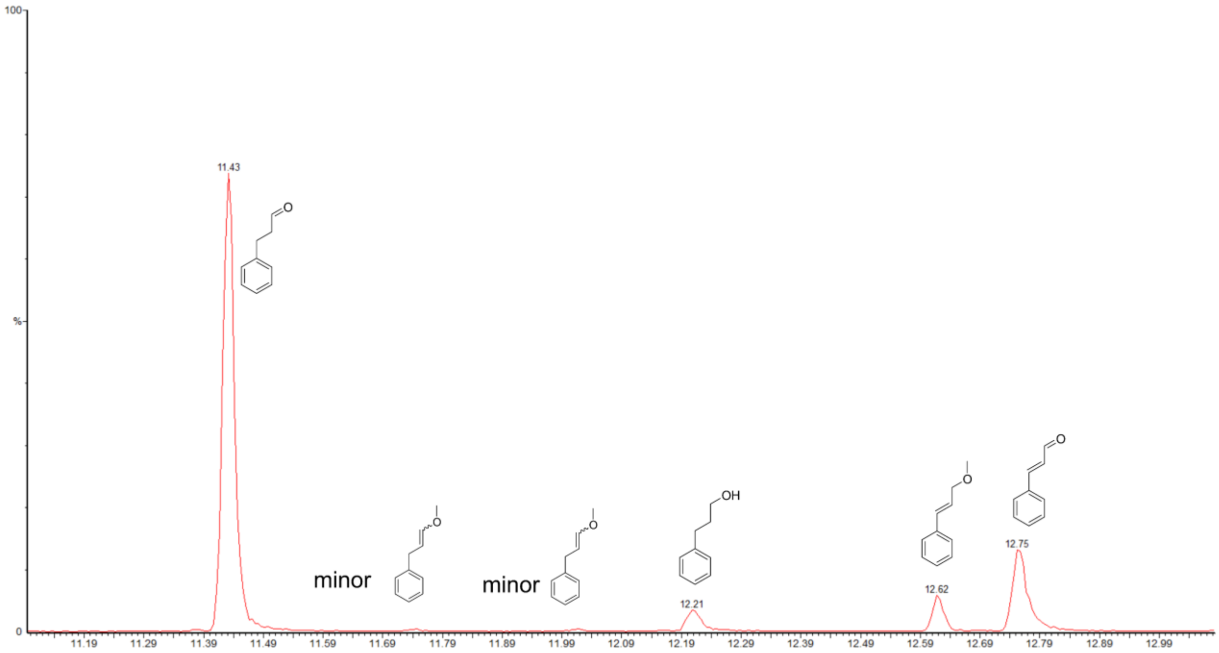

Supplementary Fig.11. GC-MS chromatogram of the reaction mixture of containing IE, cinnamyl alcohol (5 mol% relative to IE) and cinnamyl methyl ether (2.5 mol% relative to IE) with HGII (0.025 mol%), 90 °C (24 hours).

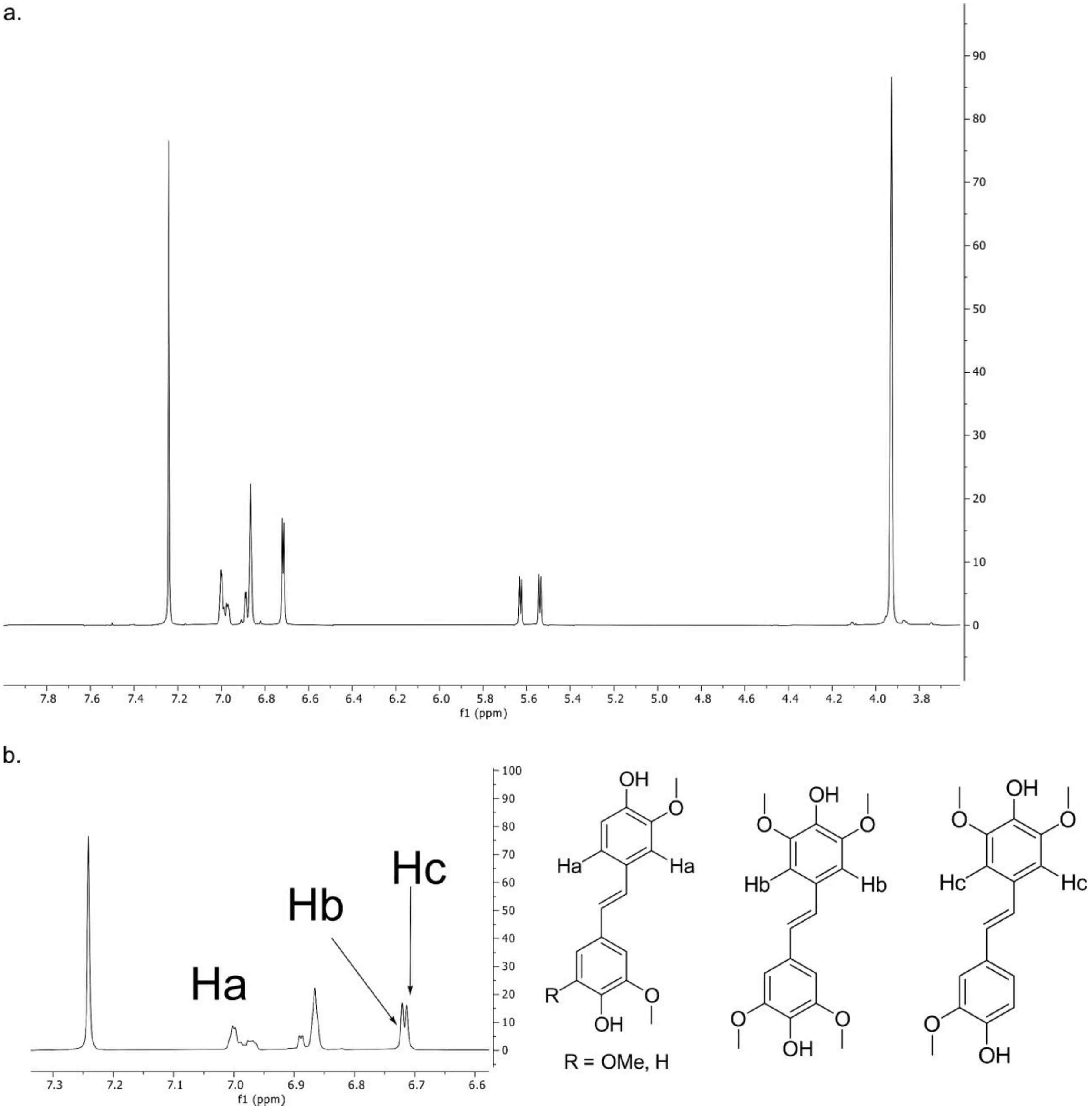

Supplementary Fig.12. Determination of the ratios of PS-PS/PS-IE/IE-IE dimers in the reaction mixtures obtained via olefin metathesis of mixtures containing PS and IE. a. An example of a full ^{1}H NMR spectrum of the reaction mixture ($CDCl_3$). b. A partial ^{1}H NMR spectrum of the reaction mixture (6.5-7.4 ppm) indicating peaks corresponding to PS-PS, PS-IE and IE-IE dimers. The deconvolution of the peak at 6.7-6.72 allowed us to obtain integrals for Hb and Hc protons (Ib and Ic respectively). An integration of the peak at 6.95-7.1 ppm allowed to obtain an integral for Ha (Ia). The molar ratio PS-PS/PS-IE/IE-IE was calculated as follows: (Ib/4)/(Ic/2)/((Ia-Ic)/4).

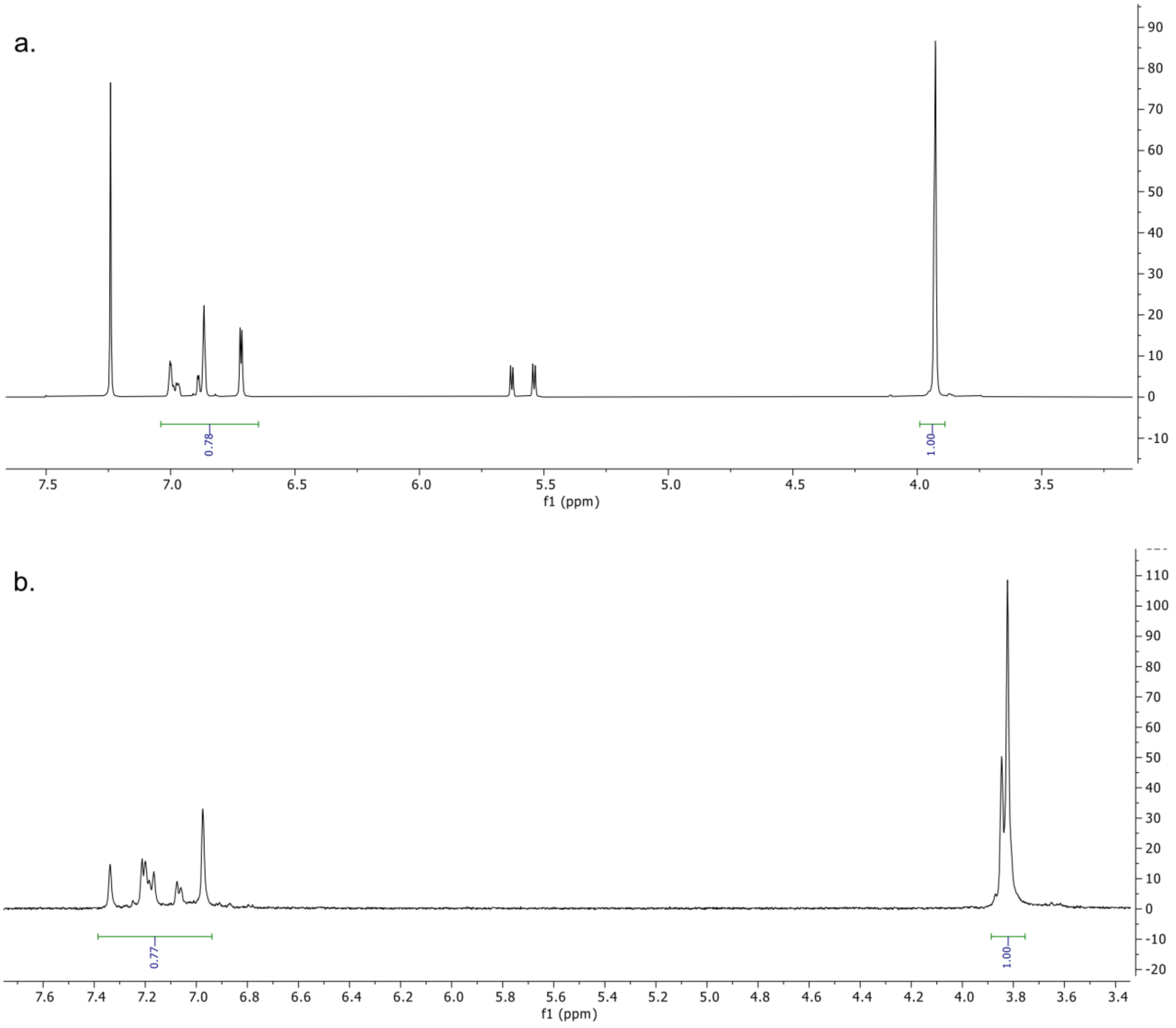

Supplementary Fig.13. a. ^{1}H NMR spectrum of the mixture of PS-PS/PS-IE/IE-IE dimers used for the preparation of P-PS-IE ($CDCl_3$). b. ^{1}H NMR spectrum of P-PS-IE (DMSO-d6).

Ratio between PS and IE units can be estimated via integration of OMe groups (3.95 ppm) and total aromatic and olefinic protons (6.68-7.1 ppm). The calculation can be performed as follows: x = mol of IE units in the starting mixture (or polymer), y = mol of PS units in the starting mixture (or polymer). x/y=R=IE/PS (mol ratio). Each IE unit contributes 4 protons to the olefinic and aromatic region (3 aromatic and 1 olefinic) and 3 protons to the methoxy group region; each PS unit contributes 3 protons to the olefinic and aromatic region (2 aromatic and 1 olefinic) and 6 protons to the methoxy group region. Based on the integration presented on the figure 12a: $(4\cdot x+3\cdot y)/(3\cdot x+6\cdot y) = 0.78$. R=1.01. Based on the integration presented on the figure 12b: R = 1.04. This indicates that upon the polymerization an initial PS/IE ratio stayed largely unchanged.

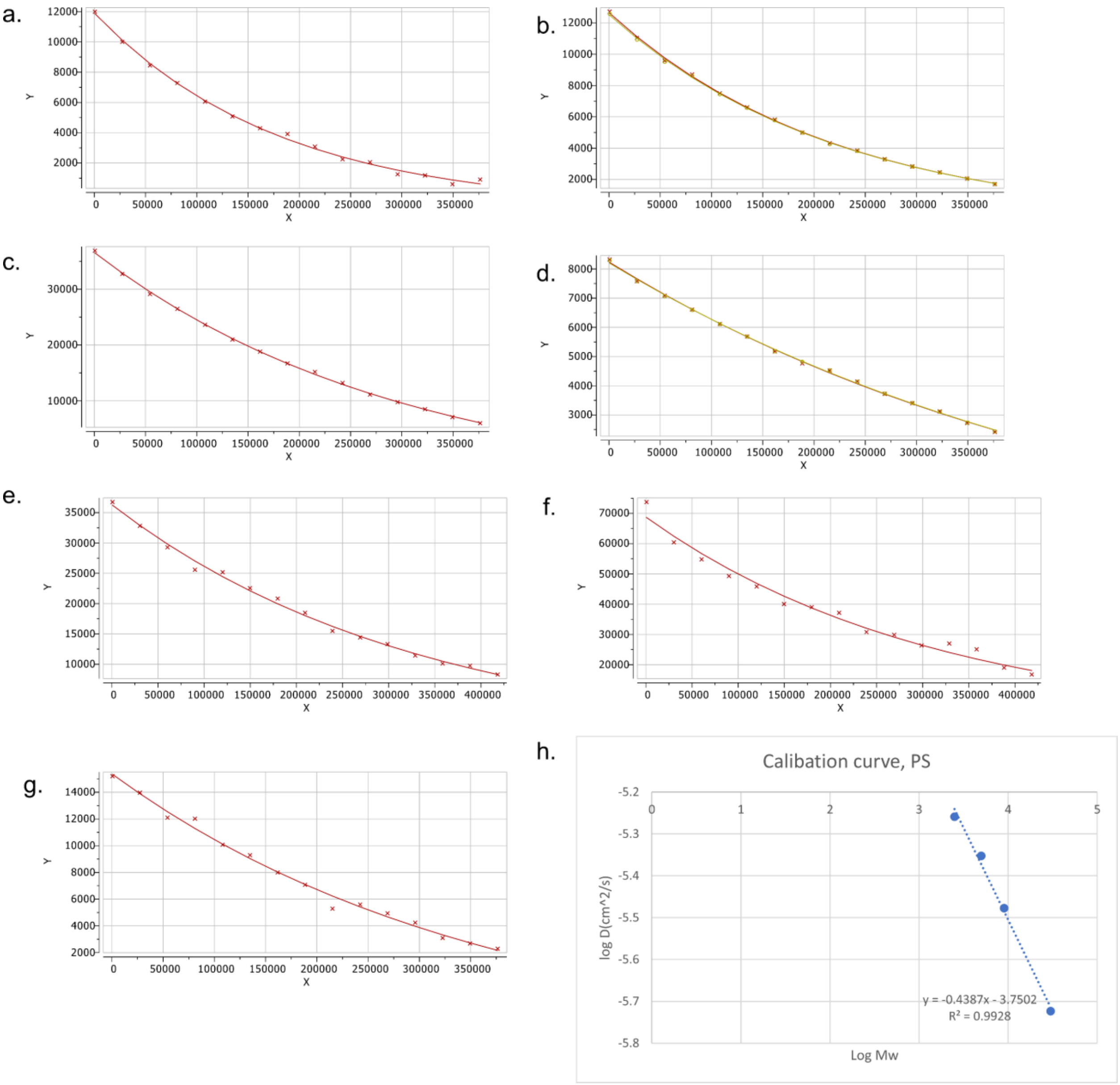

Supplementary Fig.14. Estimation of the molecular weight of the prepared polyesters by DOSY NMR (DMSO-d6). a. Decay curve for polystyrene Mw=2500. b. Decay curve for polystyrene Mw=5000. c. Decay curve for polystyrene Mw=9000. d. Decay curve for polystyrene Mw=30000. e. Decay curve for P-IE-IE. f. Decay curve for P-PS-IE . g. Decay curve for polyester prepared from lignin-derived dimers. h. Calibration curve for the molecular weight determination.

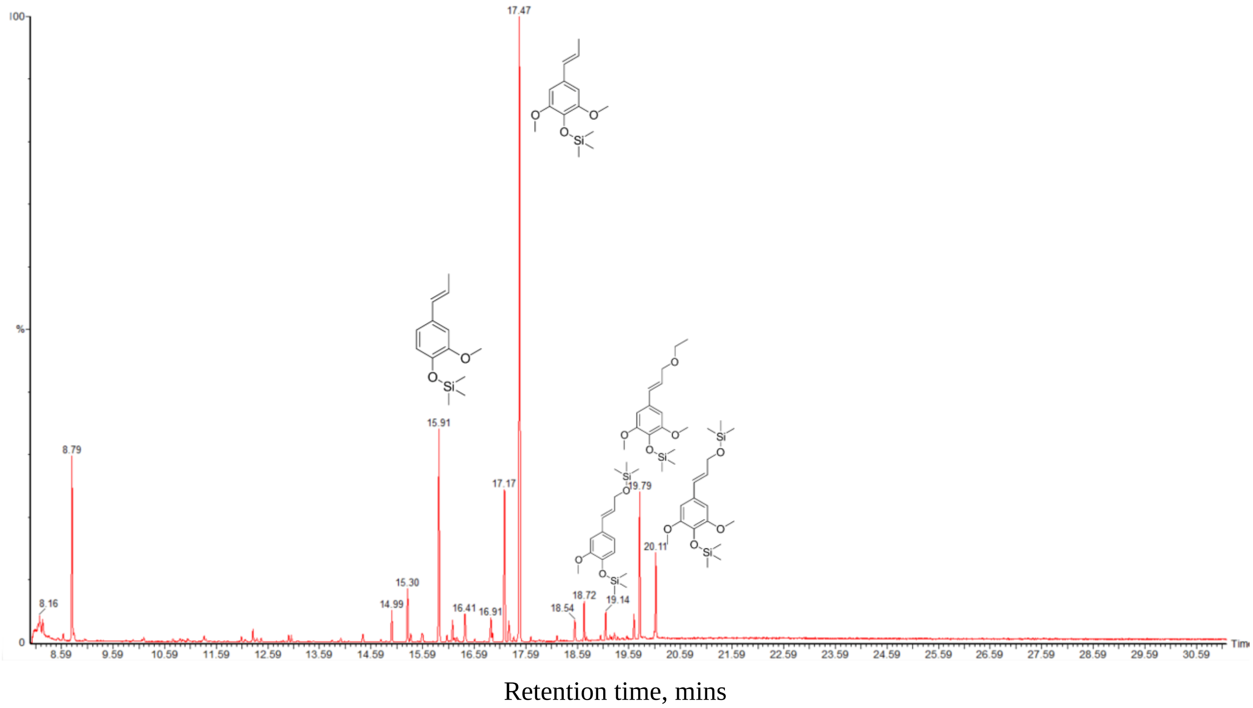

Supplementary Fig.15. GC-MS chromatogram of the silylated LO obtained at 190 °C (LO-190).

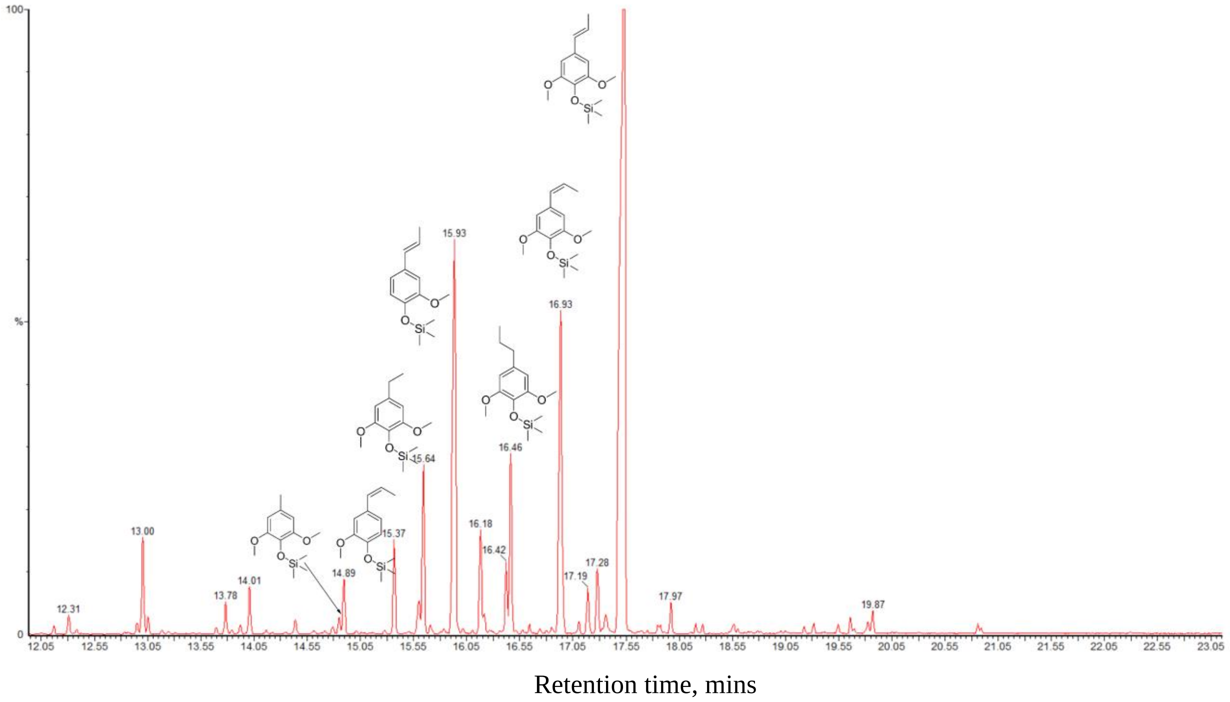

Supplementary Fig.16. GC-MS chromatogram of the silylated LO obtained at 210 °C (LO-210).

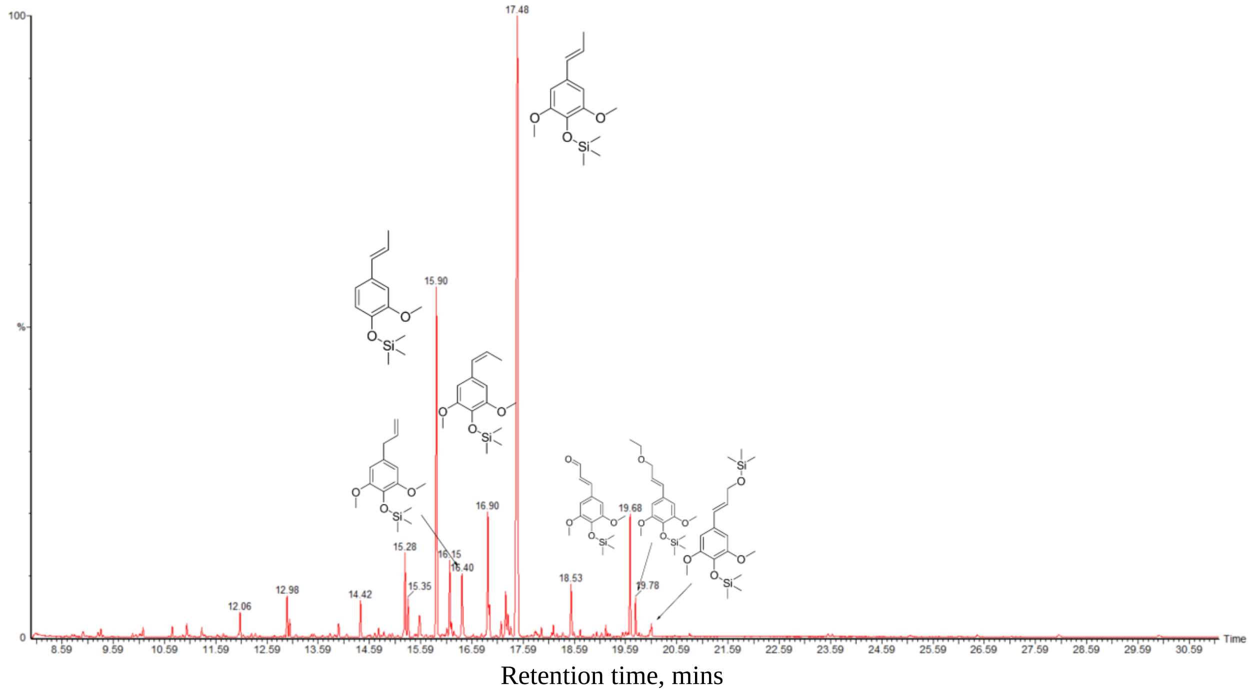

Supplementary Fig.17. GC-MS chromatogram of the silylated LO obtained at 200 °C (LO-200).

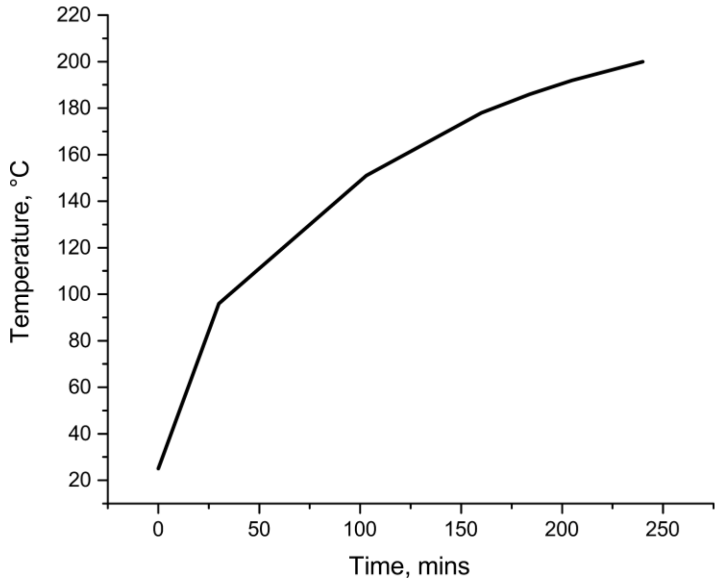

Supplementary Fig.18. An example of the heating profile during RCF.

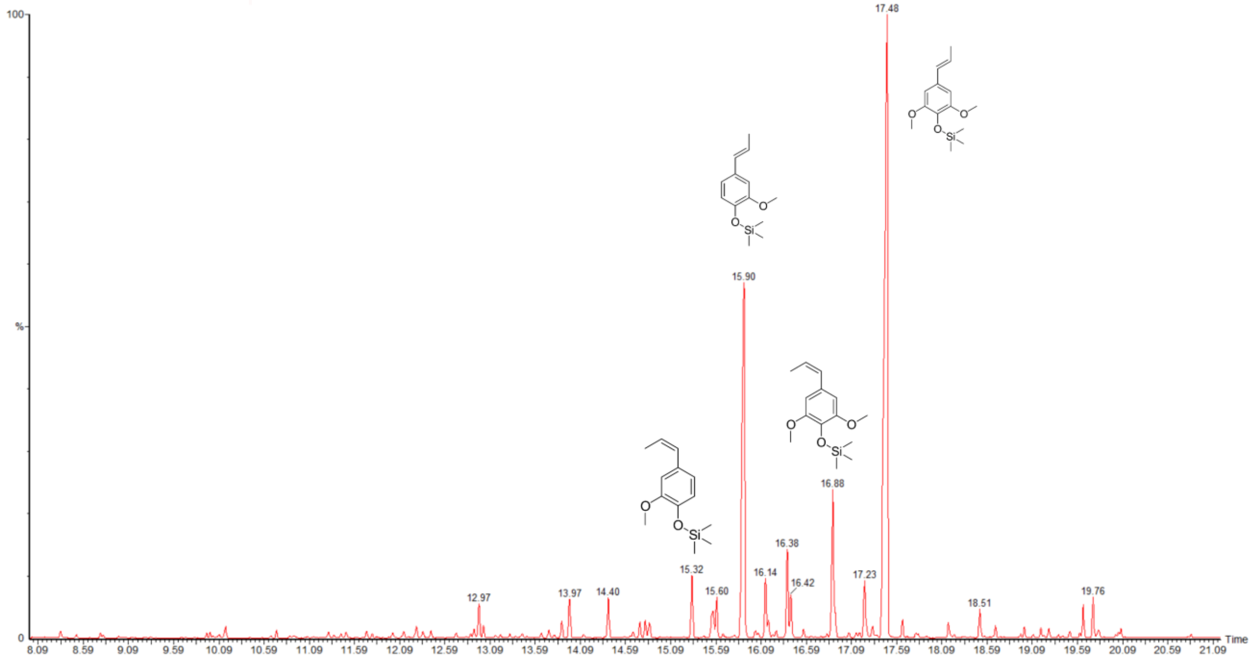

Retention time, mins

Supplementary Fig.19. GC-MS chromatogram of the silylated lignin monomers oil (LMO) obtained at 190 °C (LMO-190).

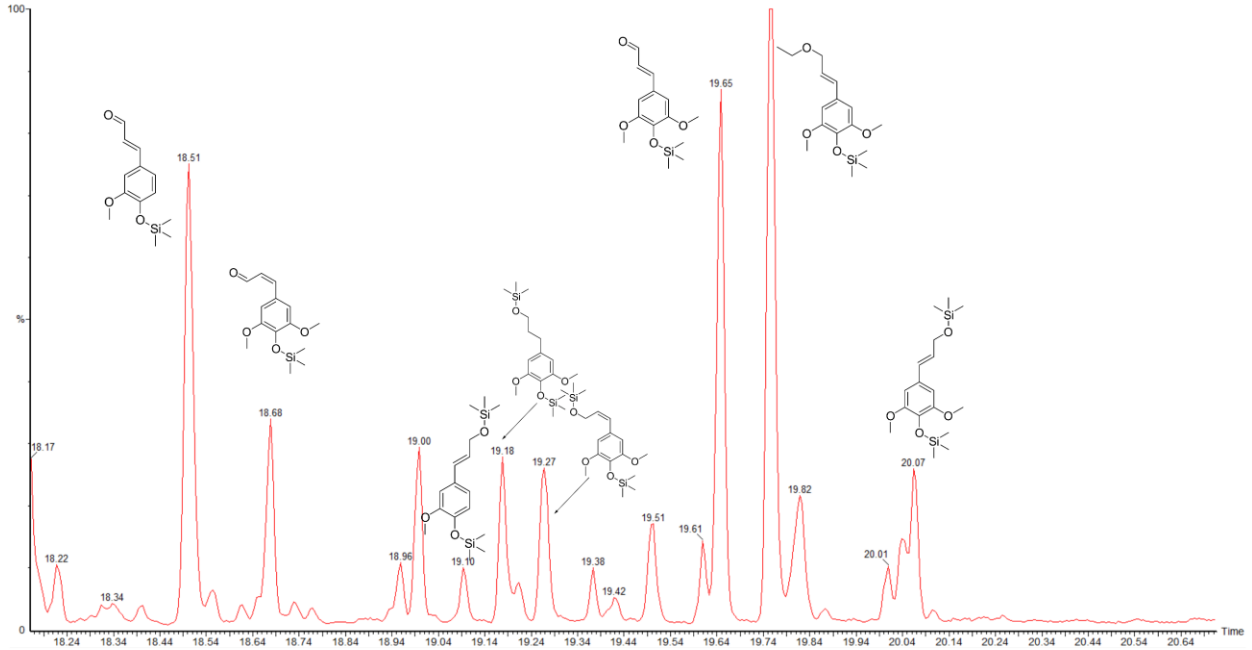

Retention time, mins

Supplementary Fig.20. Allylic region of GC-MS chromatogram of the silylated lignin monomers oil (LMO) obtained at 190 °C (LMO-190).

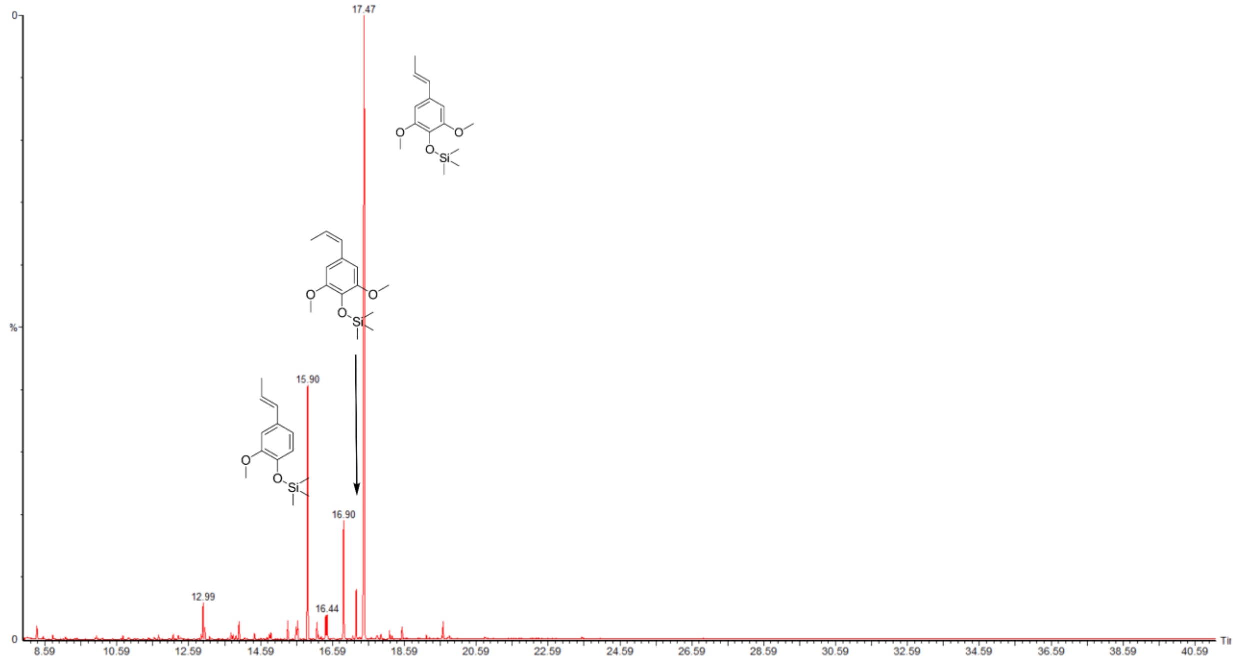

Retention time, mins

Supplementary Fig.21. GC-MS chromatogram of the silylated LMO-200.

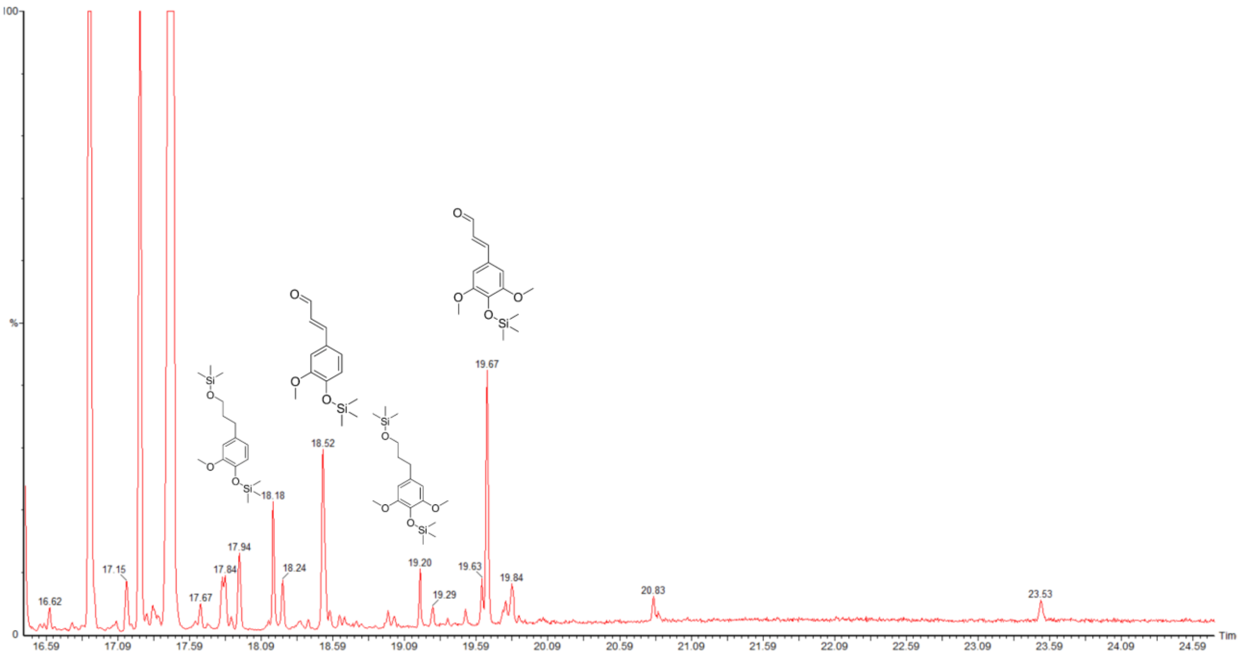

Retention time, mins

Supplementary Fig.22. Allylic region of GC-MS chromatogram of the silylated LMO-200.

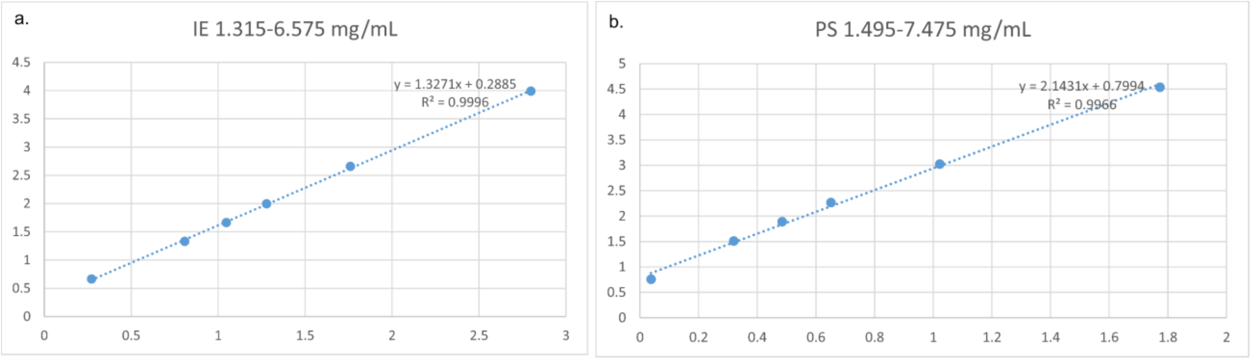

Supplementary Fig.23. Calibration curves for IE and PS obtained using GC-FID and dodecane as an internal standard.

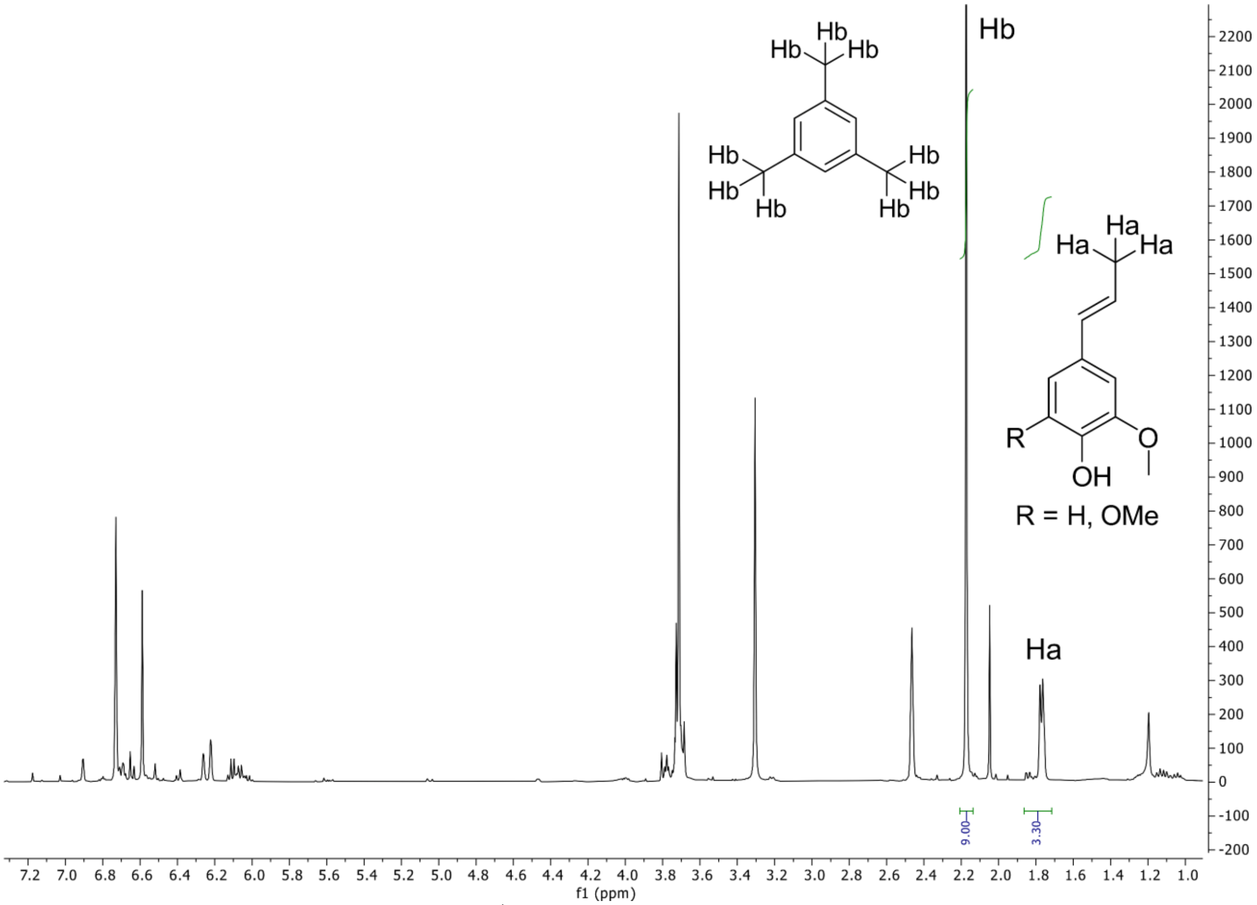

Supplementary Fig.24. An example of ^{1}H NMR spectrum for the quantification of PS and IE using mesitylene as an internal standard in DMSO-d6. The signals of methyl groups of mesitylene and methyl groups of IE and PS were used for the quantification.

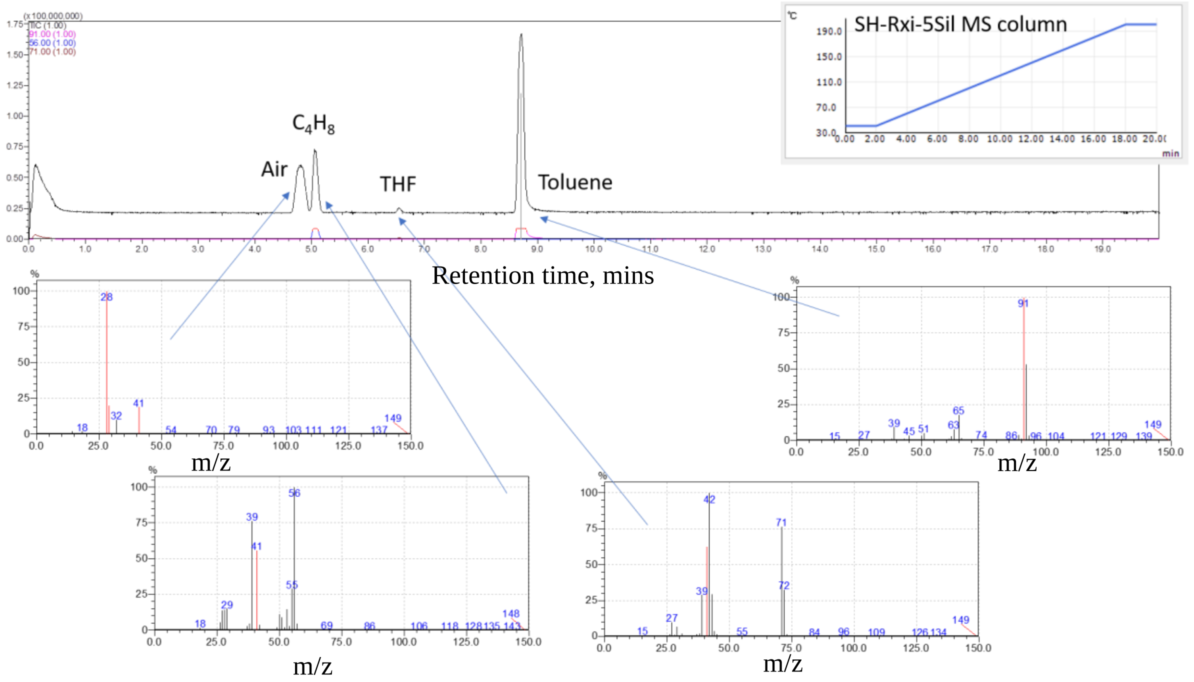

Supplementary Fig.25. GC-MS chromatogram of the gaseous phase of the metathesis reaction of LMO-200.

a.

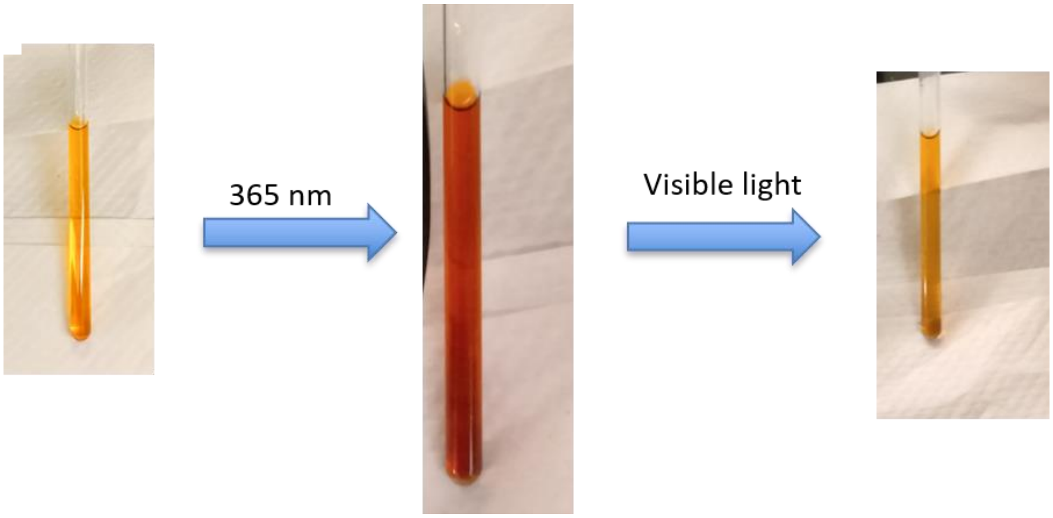

b.

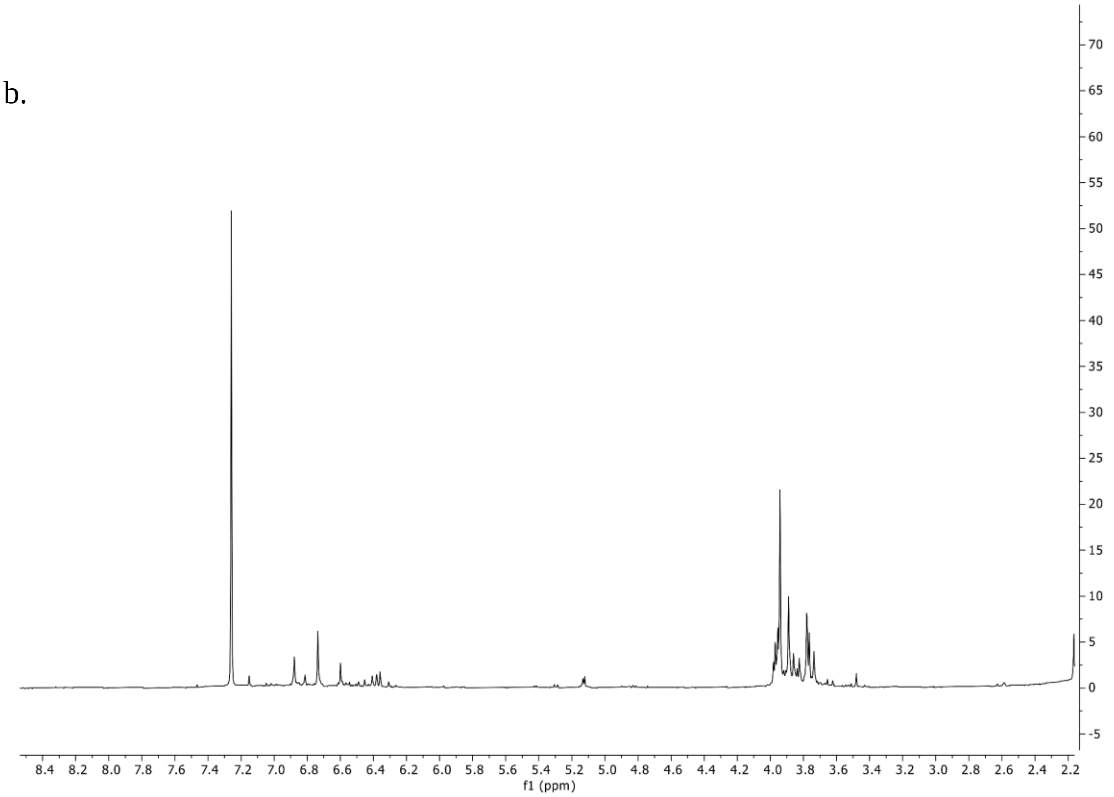

Supplementary Fig.26. Color change of the solution of lignin-derived dimers in $CDCl_3$ upon exposure to UV light (365 nm, 5 mins) and white light (5 mins) (a). ^{1}H NMR spectrum of lignin-derived dimers after exposure to UV light (365 nm) in $CDCl_3$ for 10 mins (b).

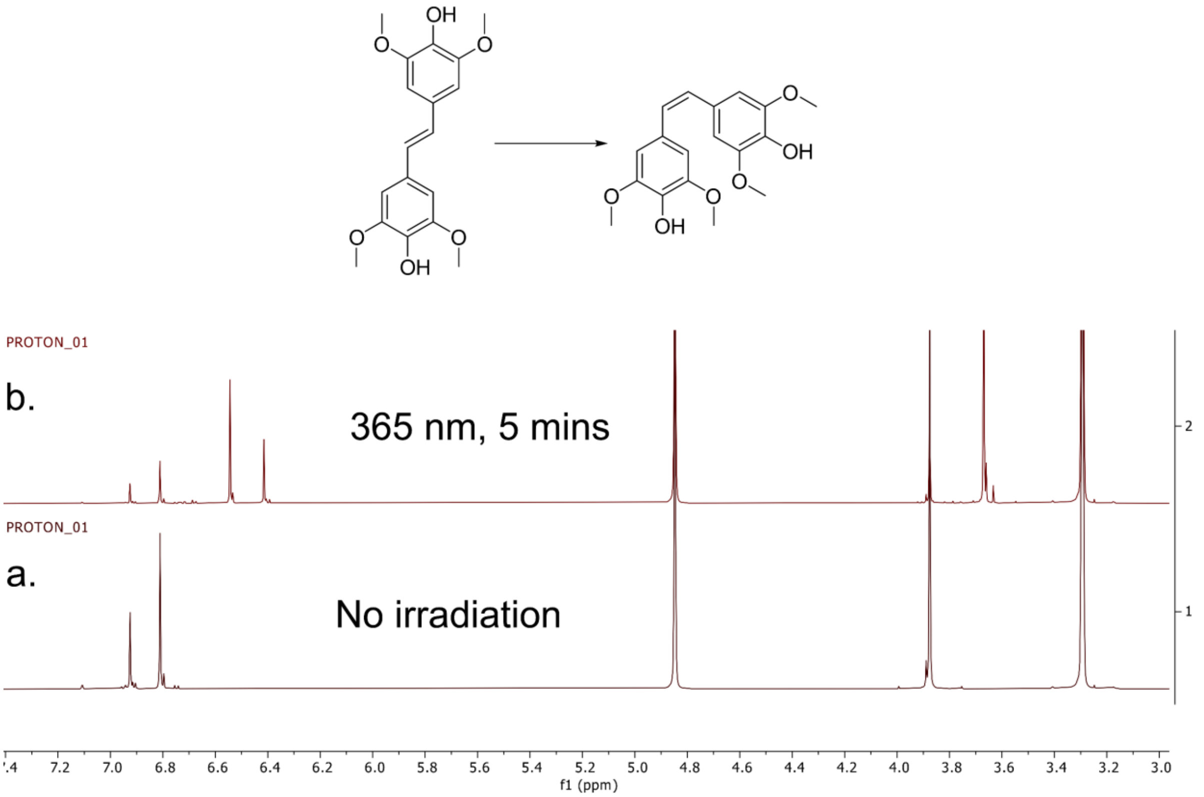

Supplementary Fig.27. ^{1}H NMR spectra of lignin-derived dimers in MeOH-d4 (a) and the spectra after exposure to UV light (365 nm) for 5 mins.

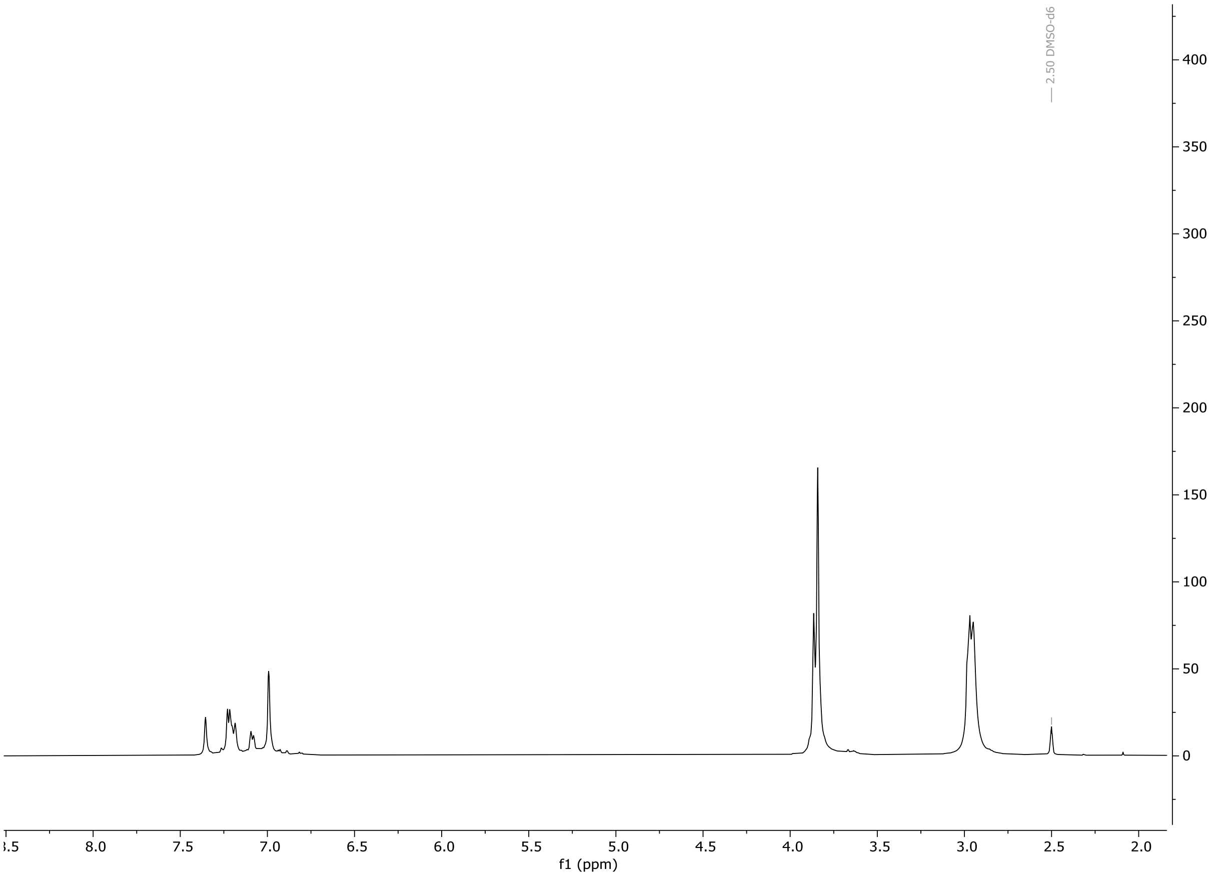

Supplementary Fig.28. ^{1}H NMR of P-PS-IE (DMSO-d6).

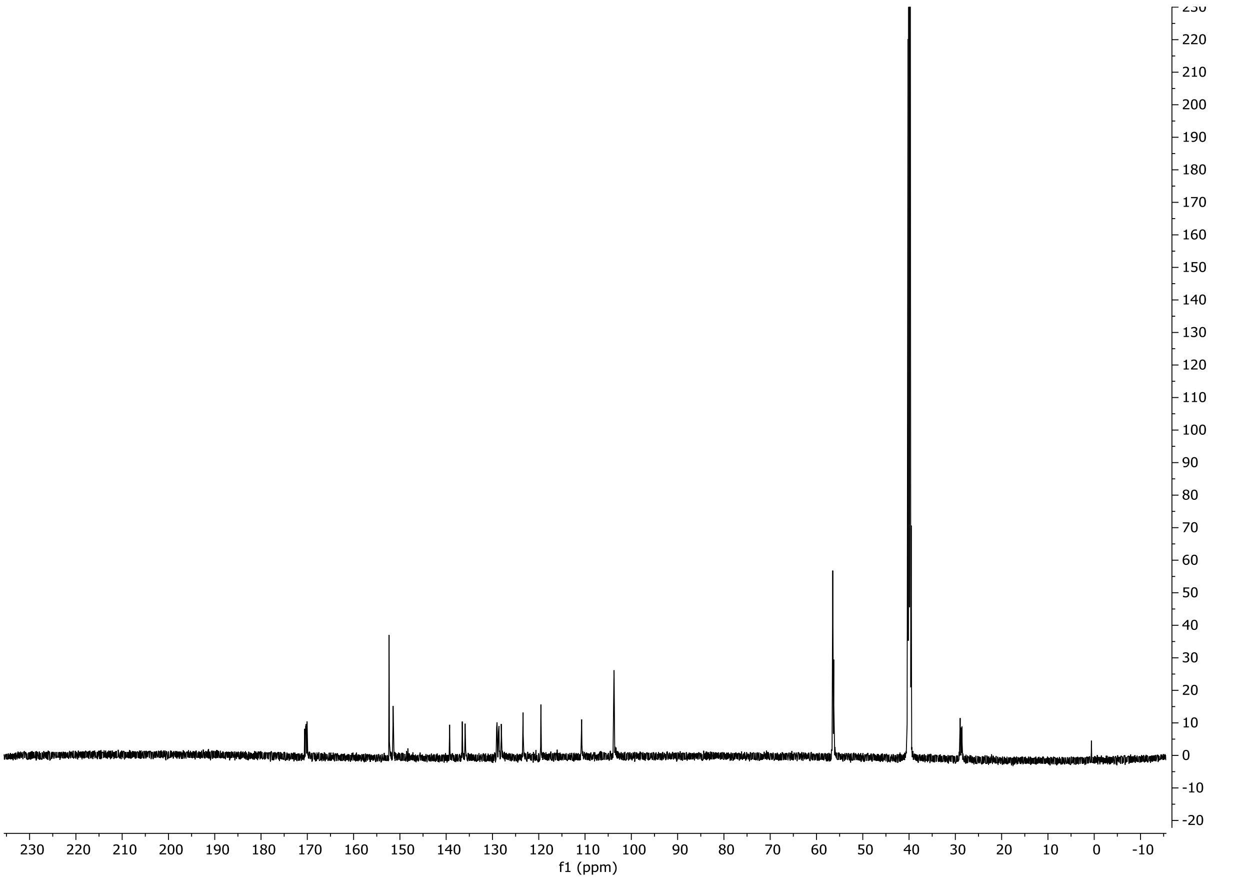

Supplementary Fig.29. ^{13}C NMR of P-PS-IE (DMSO-d6).

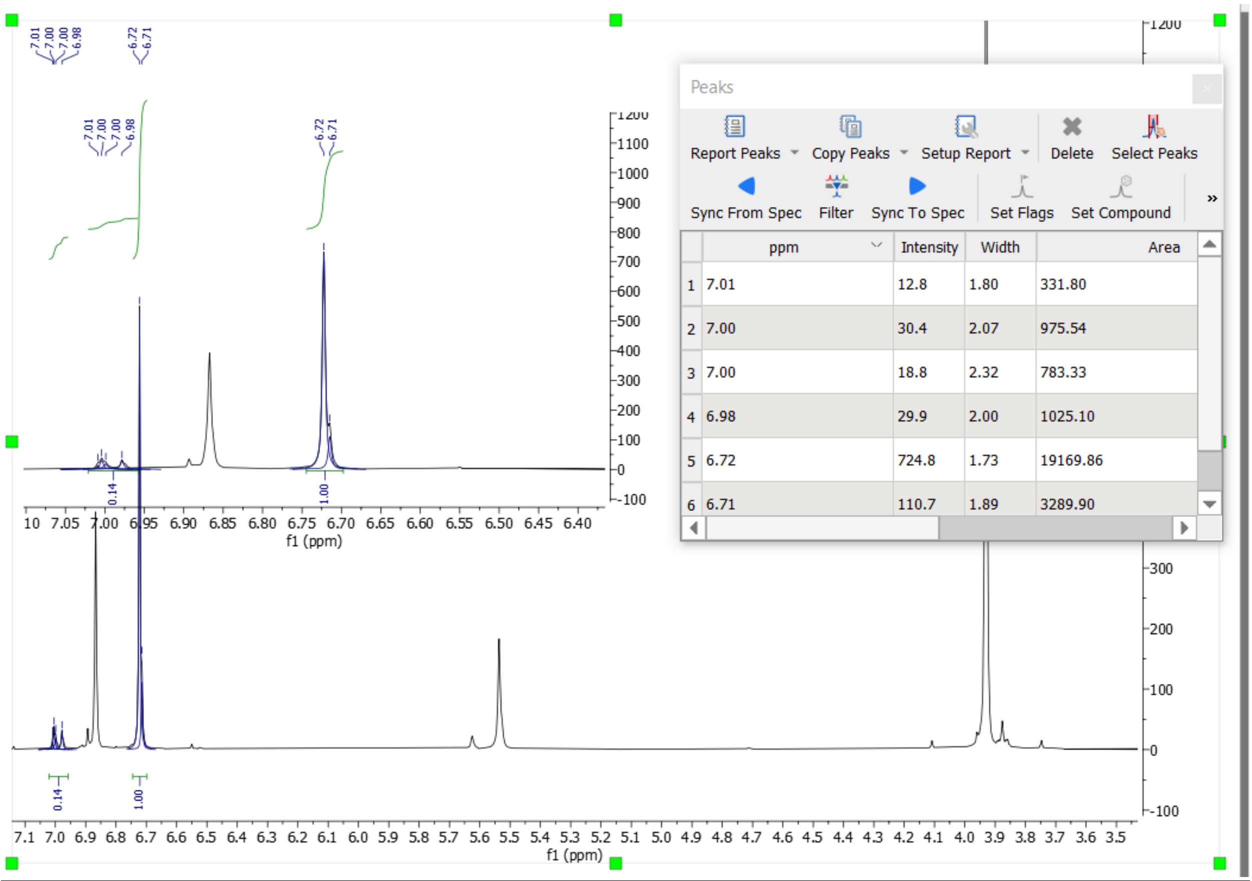

Supplementary Fig.30. ^{1}H NMR spectrum ($CDCl_3$) of mixture of LMO dimers used for the preparation of polyesters. Calculation of PS-PS/PS-IE/IE-IE ratio was performed as described on the caption for Supplementary Fig. 12.

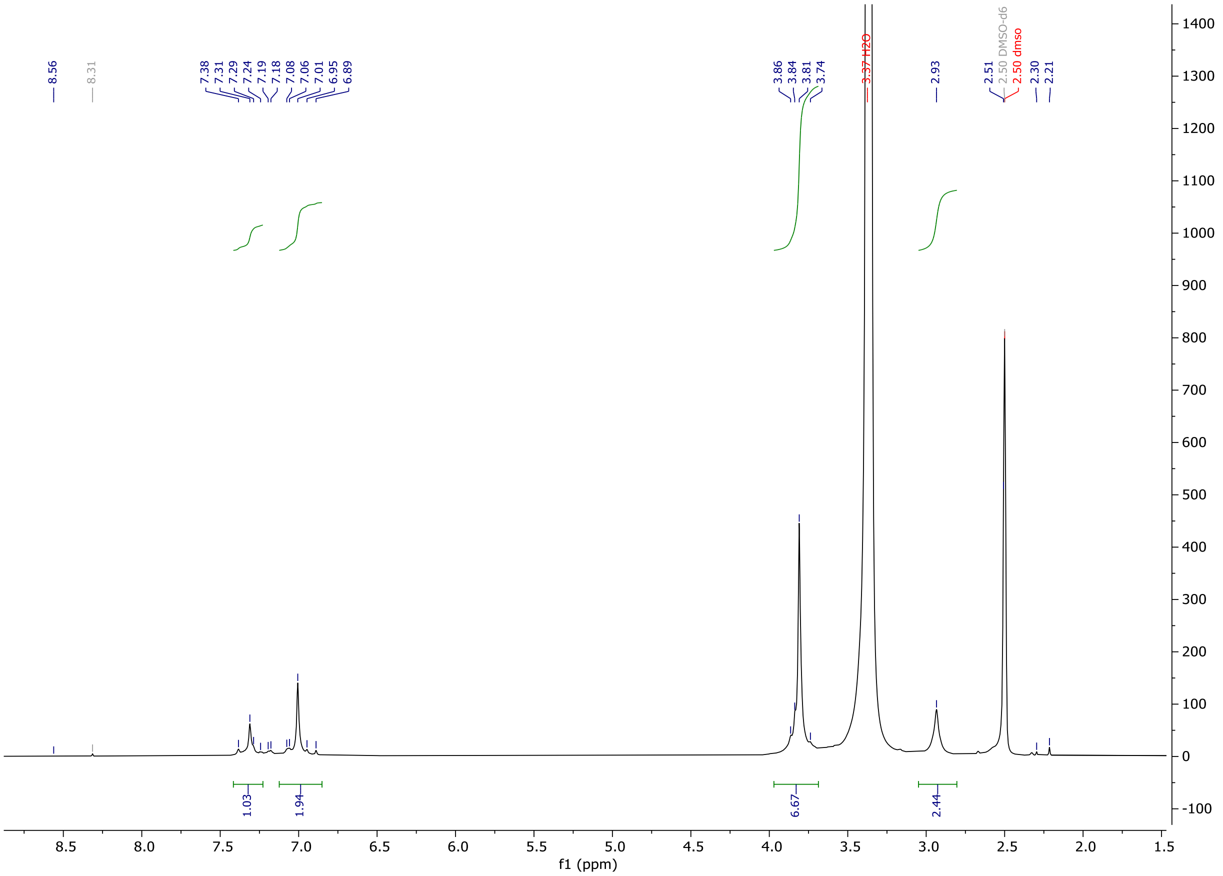

Supplementary Fig.31. ^{1}H NMR of the polyester prepared from LMO dimers (DMSO-d6).

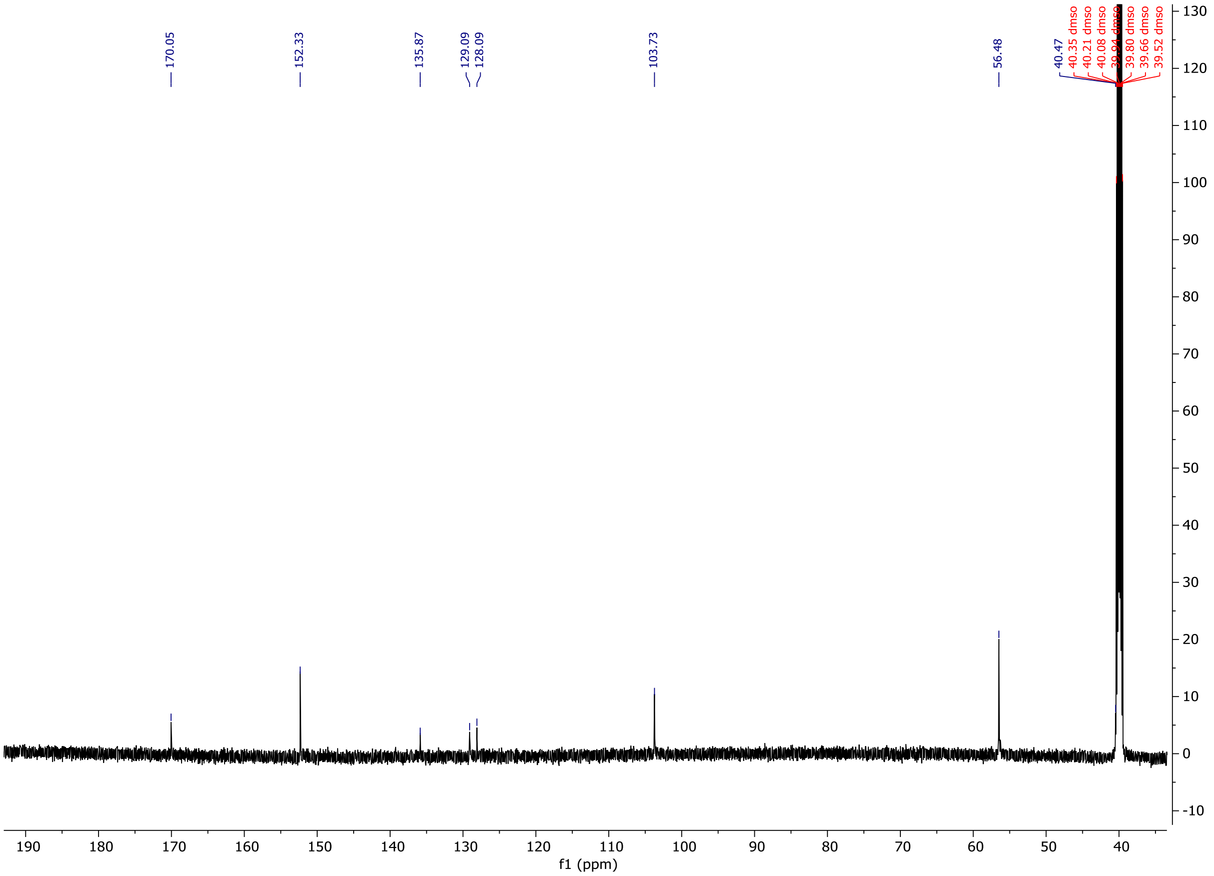

Supplementary Fig.32. ^{13}C NMR of the polyester prepared from LMO dimers (DMSO-d6).

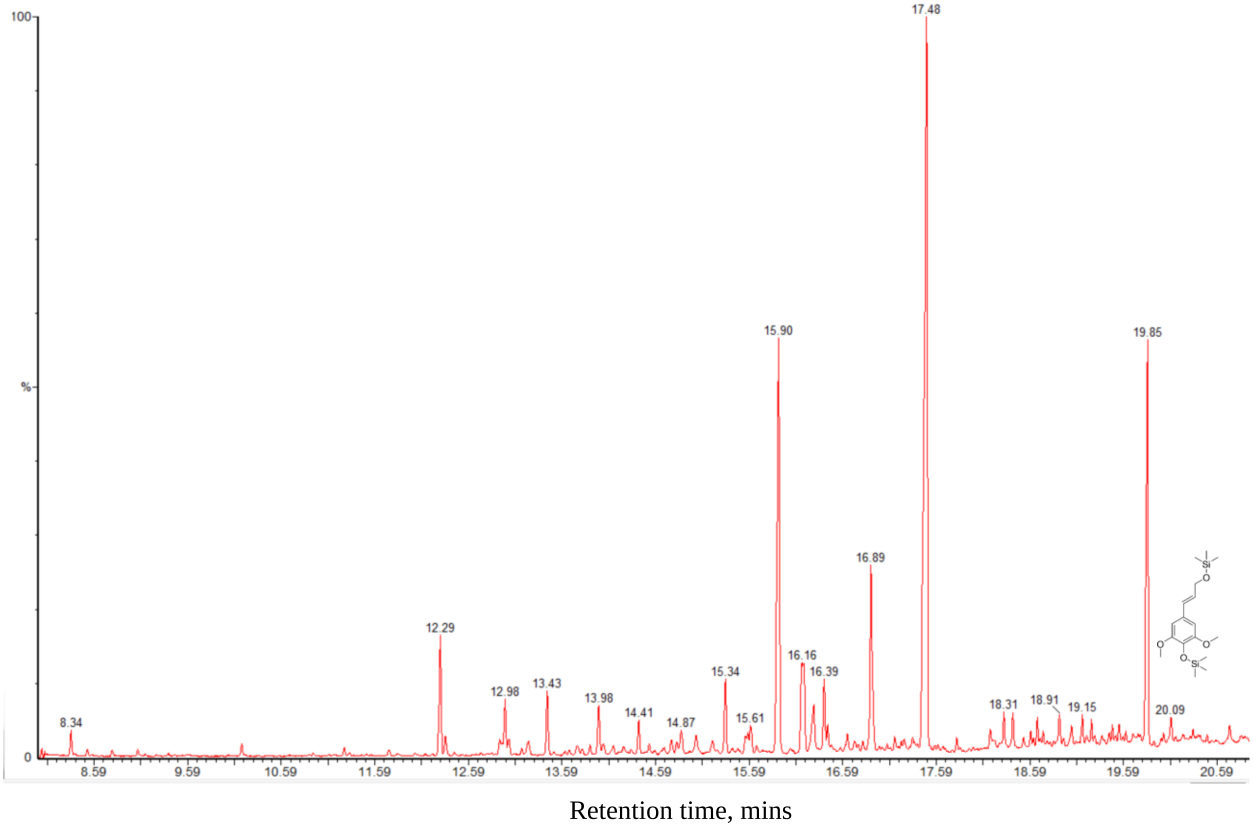

Supplementary Fig.33. GC-MS chromatogram of the lignin monomers oil (LMO) obtained at 200 °C from non-sieved wood.

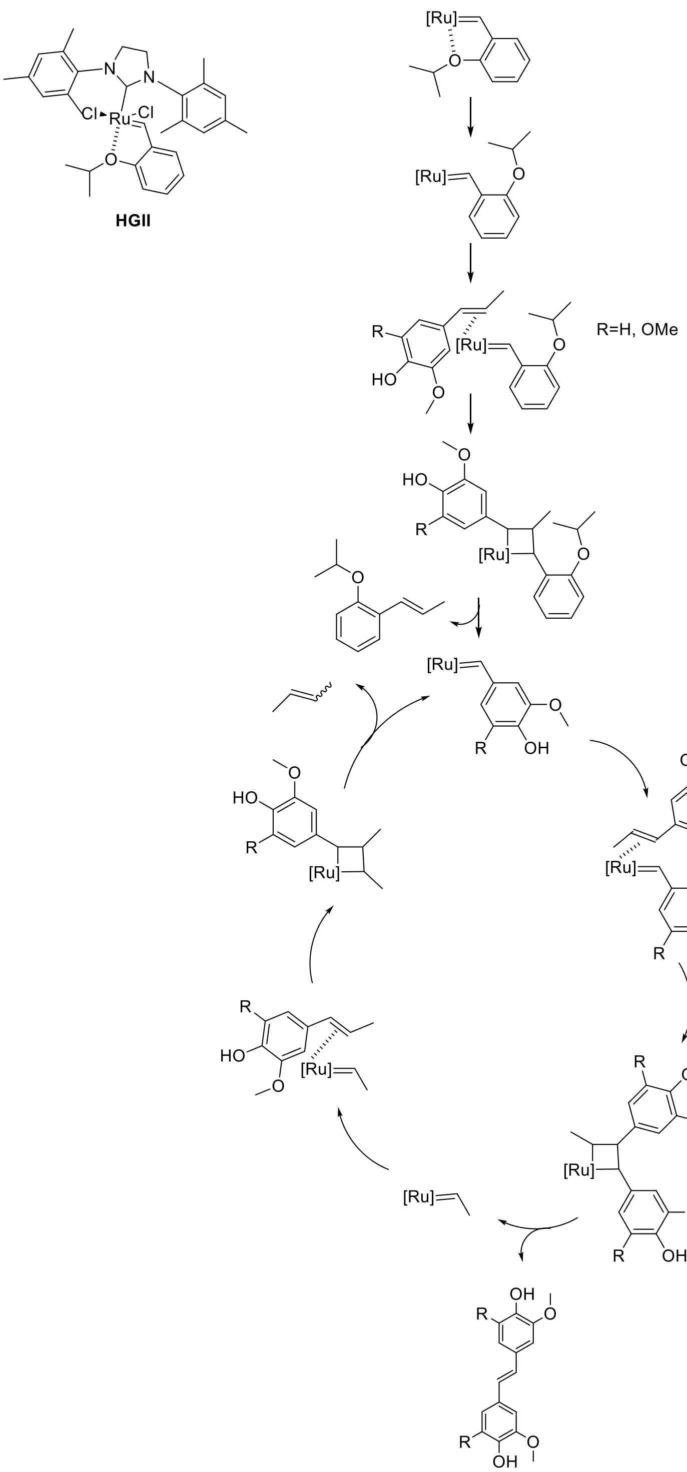

Supplementary Fig.34. A general reaction mechanism for the olefin metathesis of IE and PS.

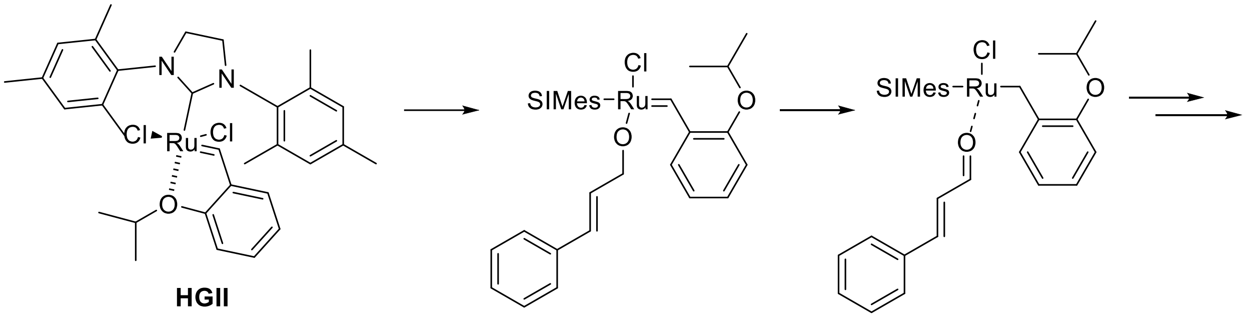

Supplementary Fig.35. A possible decomposition pathway of HGII via dehydrogenation of cinnamyl alcohol.

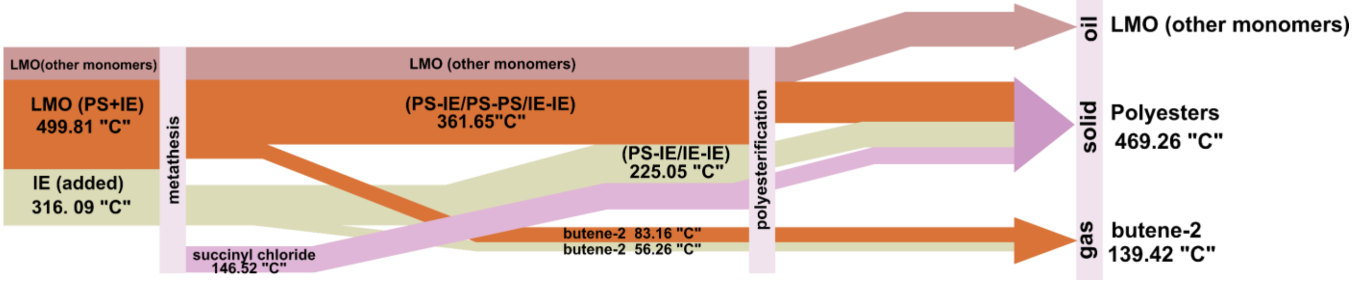

Supplementary Fig.36. Carbon flow analysis. The calculations are performed using the following assumptions: 1000 tons of LMO containing 72 wt% of PS and IE (PS/IE molar ratio 2.3) with addition of 60 wt% of IE relative to the total IE+PS content in LMO; 89% yield of the bisphenols in metathesis step; 64% yield of the polyesters during the polyesterification step (1.05 equiv. of succinyl chloride). The numbers on the diagram correspond to total carbon mass in the corresponding product.

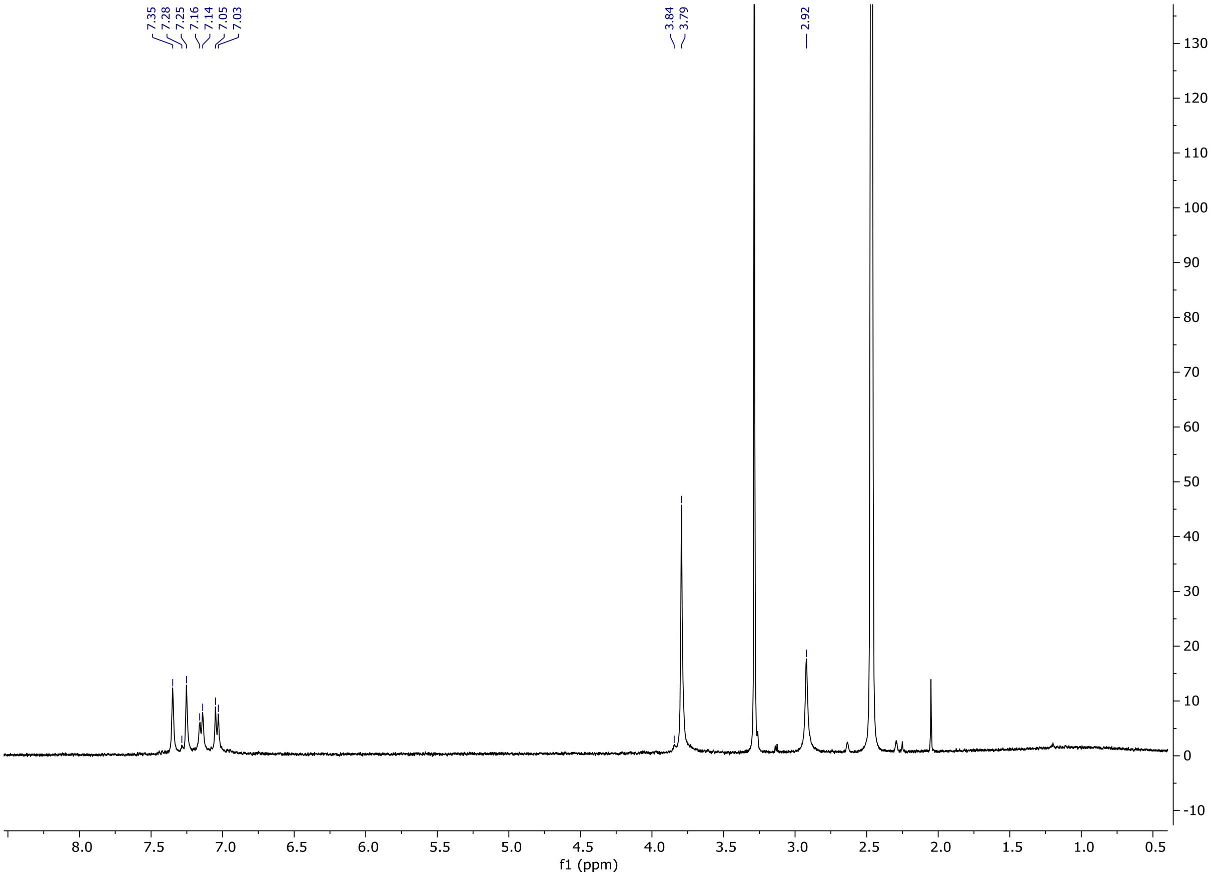

Supplementary Fig.37. ^{1}H NMR spectrum of P-IEIE.

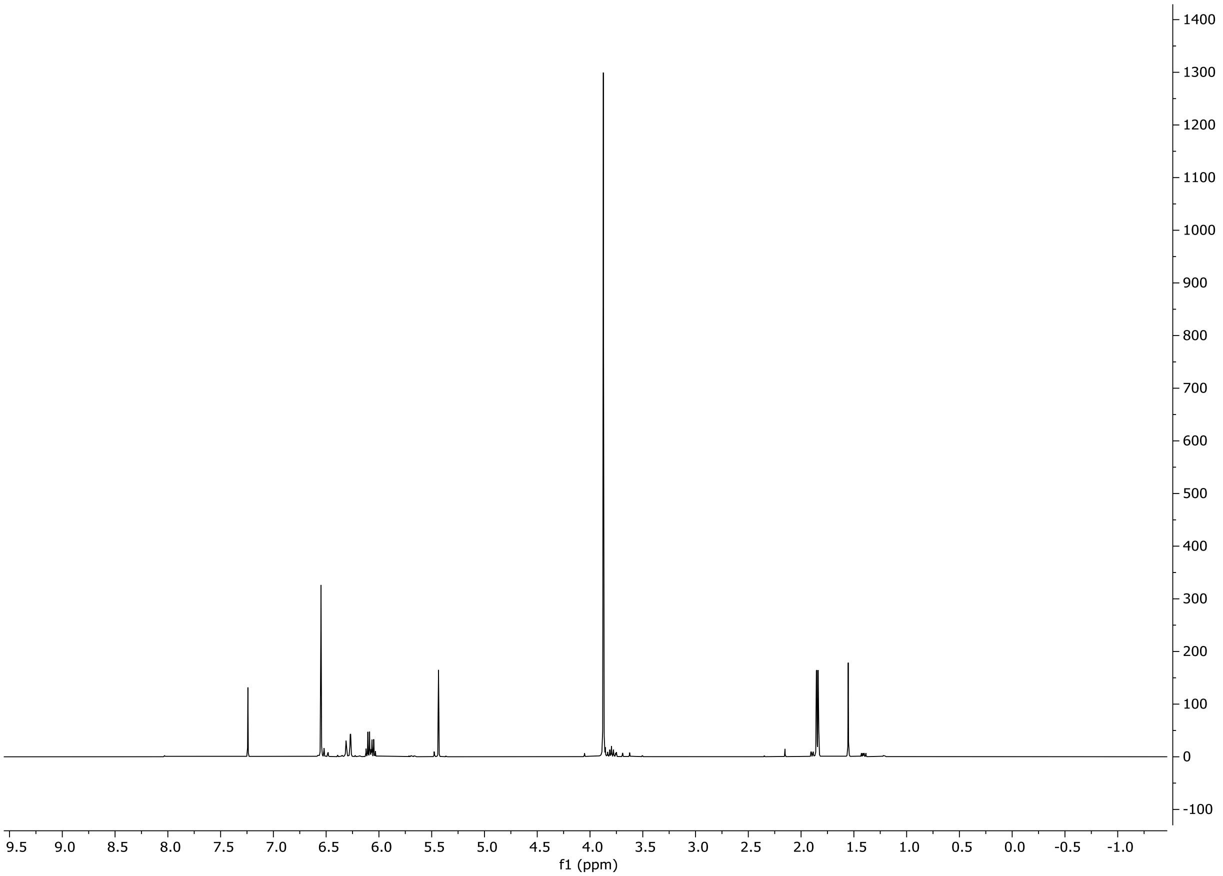

Supplementary Fig.38. ^{1}H NMR spectrum of PS.

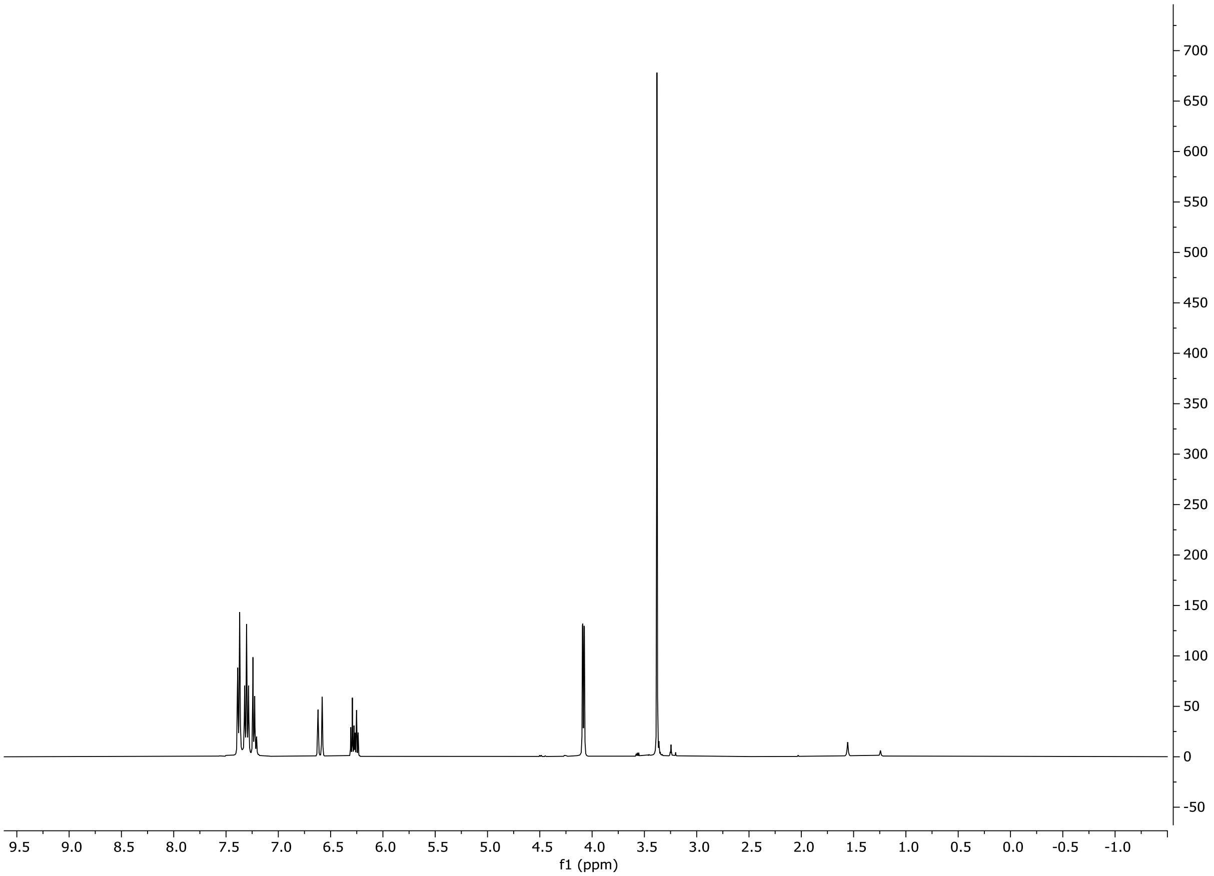

Supplementary Fig.39. ^{1}H NMR spectrum of methyl cinnamyl ether.

3. Supplementary Tables

Supplementary Table 1. Yield of lignin oil (LO) from RCF of wood at different temperatures.

RCF temp, °C	Reaction time*, h	Yield of LO, wt%
190	2	59.5
200	2	72.8
200	4	95
210	2	100

* Reaction time after the final temperature was reached.

Supplementary Table 2. Quantitative analysis of the major identified compounds in LMO-200.

Retention time, mins	m/z	Proposed structure
12.99	198, 183, 169, 155, 139,111, 109, 99, 91, 81, 75, 73, 61	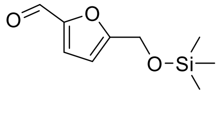

13.91	224, 209, 194, 179, 149, 73	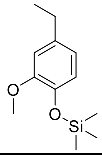
13.99	268, 226, 211, 196, 181, 153, 73	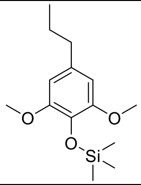
14.42	222, 207, 192, 177, 162, 147, 73	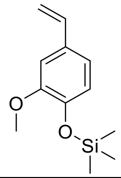
14. 77	236, 221, 206, 179, 73	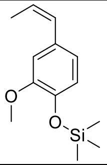
14.83	238, 209, 179, 149, 73	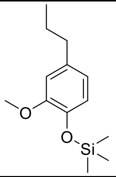
15.34	236, 206, 73	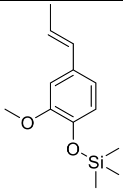
15.62	254, 239, 209, 73	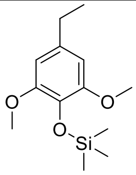
15.88	236, 206, 73	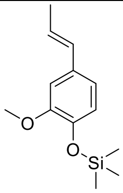
16.15	252, 237, 222, 179, 73	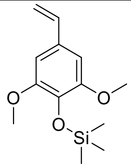

16.40	266, 251, 236, 221, 205, 73	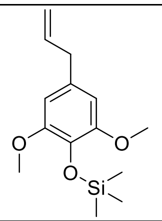
16.44	268, 253, 238, 209, 73	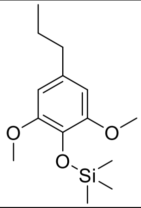
16.90	266, 236, 205, 73	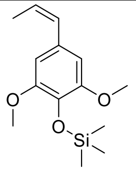
17.47	266, 251, 236, 221, 205, 73	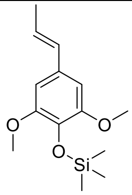
18.18	326, 311, 236, 206, 192, 179, 73	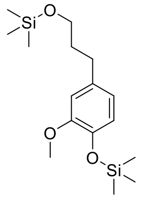
18.52	250, 235, 220, 192, 73	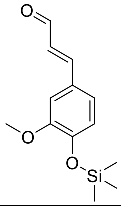
19.20	356, 341, 240, 210, 73	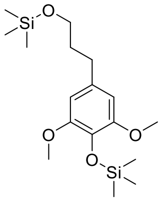
19.67	280, 265, 250, 222, 207, 179, 73	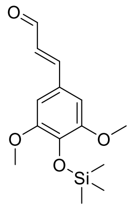

4. Python script

Example of the python script for calculation of the ratio of IEIE(aa)/PSPS(bb)/PSIE(ab) dimers for random coupling during olefin metathesis (ratio of PS to IE 3/1).

```
"create an empty list of n elements"

monomers = [None]*10000

"fill the list with letters a and b (number of PS and IE fragments)"

for i in range (0, 2500):
    monomers[i] = "a"
for i in range (2500, 10000):
    monomers[i] = "b"

"aa, ab, bb correspond to number of hetero and homo dimers"

aa = 0
bb = 0
ab = 0

"choose and remove 2 random elements (random_1 and random_2) from monomers"

import random
while len(monomers) > 0:
    random_1 = random.choice(monomers)
    random_2 = random.choice(monomers)
    monomers.remove(random_1)
    monomers.remove(random_2)

    " count dimers aa, bb and ab"
      if random_1 == "a" and random_2 == "a" :
        aa = aa+1
        bb = bb
        ab = ab
    elif random_1 == "b" and random_2 == "b":
        bb = bb+1
        aa = aa
        ab =ab
    else:
        ab = ab+1
        aa = aa
        bb = bb
print (aa, bb, ab)
```

Supplementary References

1. Patil SN, Tilve SG. Concise access toward chiral hydroxy phenylpropanoids: formal synthesis of virolongin B; kigelin; kurasoin A; 4-hydroxysattabacin, and actinopolymorphol A. *Tetrahedron Lett* **57**, 3371-3375 (2016).

2. Straathof NJW, Cramer SE, Hessel V, Noël T. Practical Photocatalytic Trifluoromethylation and Hydrotrifluoromethylation of Styrenes in Batch and Flow. *Angew Chem Int Ed* **55**, 15549-15553 (2016).
